# Blue light induces apoptosis and autophagy by promoting ROS‐mediated mitochondrial dysfunction in synovial sarcoma

**DOI:** 10.1002/cam4.5664

**Published:** 2023-02-01

**Authors:** Makoto Takeuchi, Toshihiko Nishisho, Shunichi Toki, Shinji Kawaguchi, Shunsuke Tamaki, Takeshi Oya, Yoshihiro Uto, Toyomasa Katagiri, Koichi Sairyo

**Affiliations:** ^1^ Department of Orthopedics Institute of Biomedical Sciences, Tokushima University Graduate School Tokushima Japan; ^2^ Department of Molecular Pathology Institute of Biomedical Sciences, Tokushima University Graduate School Tokushima Japan; ^3^ Graduate School of Technology, Industrial and Social Sciences Tokushima University Tokushima Japan; ^4^ Division of Genome Medicine Institute of Advanced Medical Sciences, Tokushima University Tokushima Japan

**Keywords:** apoptosis, autophagy, blue light, mitochondria, reactive oxygen species, synovial sarcoma

## Abstract

**Background:**

Synovial sarcoma (SS) has limited treatment options and there is an urgent need to develop a novel therapeutic strategy to treat SS. Blue light (BL) has been shown to inhibit the growth of several cancer cells. However, the efficacy of BL in soft tissue sarcomas such as SS has not been demonstrated, and the detailed mechanism underlying the antitumor activity of BL is not fully understood. In this study, we investigated the antitumor effect of BL on SS.

**Methods:**

Human SS cell lines were continuously irradiated with BL using light‐emitting diodes (LEDs) in an incubator for in vitro analysis. The chicken chorioallantoic membrane (CAM) tumors and xenograft tumors in mice were subjected to daily BL irradiation with LEDs.

**Results:**

BL caused growth inhibition of SS cells and histological changes in CAM tumors. BL also suppressed the migration and invasion abilities of SS cells. The type of cell death in SS cells was revealed to be apoptosis. Furthermore, BL induced excessive production of reactive oxygen species (ROS) in mitochondria, resulting in oxidative stress and malfunctioned mitochondria. Reducing the production of ROS using N‐acetylcysteine (NAC), a ROS scavenger, attenuated the inhibitory effect of BL on SS cells and mitochondrial dysfunction. In addition, BL induced autophagy, which was suppressed by the administration of NAC. The autophagy inhibitor of 3‐methyladenine and small interfering RNA against the autophagy marker light chain 3B facilitated apoptotic cell death. Moreover, BL suppressed tumor growth in a mouse xenograft model.

**Conclusion:**

Taken together, our results revealed that BL induced apoptosis via the ROS‐mitochondrial signaling pathway, and autophagy was activated in response to the production of ROS, which protected SS cells from apoptosis. Therefore, BL is a promising candidate for the development of an antitumor therapeutic strategy targeting SS.

## Graphical abstract

In this study, we investigated the antitumor effect of Blue light (BL) on Synovial sarcoma (SS). Here, we found that BL induced apoptosis via the ROS‐mitochondrial signaling pathway, and autophagy was activated in response to the production of ROS, which protected SS cells from apoptosis. Therefore, BL is a promising candidate for the development of an antitumor therapeutic strategy targeting SS.

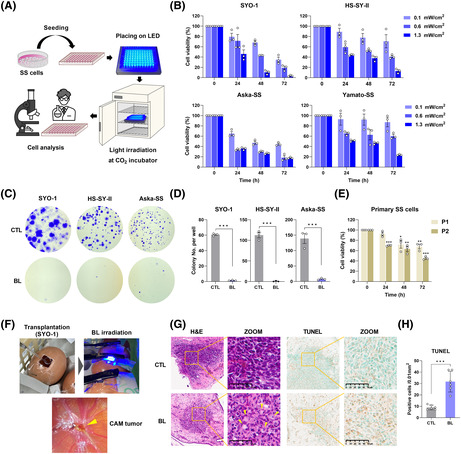

## 1 INTRODUCTION

Synovial sarcoma (SS) is a highly malignant soft tissue tumor that can arise in various parts of the body, but most that tend to arise from sites near joints, including the bursae, joint capsules, and tendon sheaths. Accounting for 5%–10% of all soft tissue sarcomas, SS is the fourth most frequent soft tissue tumor.^1^ The current treatment for localized SS is surgical excision with a broad margin of the surrounding normal tissue, occasionally with combined radiotherapy and chemotherapy. The 5‐year survival rate is estimated to be 40%–60%,^2^, ^3^ but when lung metastases develop or there is recurrence of the primary tumor, the prognosis is poor, even with intensive multidrug chemotherapy. Because of the limited availability of effective treatments, there is an urgent need to develop novel therapies for patients with SS.

In the present era, humans can conveniently control visible light in a specific narrow wavelength owing to the invention and development of light‐emitting diodes (LEDs), which are a novel luminous source dating back to the late 1900s. In medicine, phototherapy using LEDs is widely used for several therapeutic targets including acne vulgaris,^4^ wound healing,^5^ skin rejuvenation,^6^ and bacterial and viral infections.^7^, ^8^ Furthermore, light irradiation at wavelengths ranging from 450 to 495 nm, which human eyes perceive as blue, has been shown to have antitumor effects on various cancer cells, including melanoma,^9^, ^10^ lymphoma,^11^ colon cancer,^12^, ^13^, ^14^, ^15^ leukemia,^16^ pancreatic cancer,^17^ and osteosarcoma.^18^ Therefore, blue light (BL) from LEDs is expected to become a novel non‐invasive therapeutic option in cancer treatment. Although the biological mechanism of BL‐induced antitumor effect is reported to be regulated by cell cycle inhibition,^9^ reactive oxygen species,^18^, ^19^, ^20^, ^21^ apoptosis,^9^, ^12^, ^17^, ^20^, ^22^ and autophagy,^11^, ^12^, ^14^, ^18^ its role and precise mechanisms remain unclear. Despite the beneficial effects of BL on various cancers, to date, the efficacy and biological response to BL in soft tissue sarcoma including SS has yet to be determined.

In this study, we investigated the ability of BL to inhibit growth in SS cells in vitro and in vivo and elucidated the underlying mechanisms. Our findings provide direct evidence that BL might exert antitumor effects on SS and may therefore be a novel treatment option for SS.

## 2 MATERIALS AND METHODS

### 2.1 Cell culture

The human SS cell lines SYO‐1, HS‐SY‐II, Aska‐SS, and Yamato‐SS as well as the human embryonic kidney cell line HEK293 were used. SYO‐1 was donated by Dr. Akira Kawai (National Cancer Center Hospital),^23^ while HS‐SY‐II, Aska‐SS, Yamato‐SS, and HEK293 were purchased from the RIKEN BioResource Center Cell Bank. SS cells from surgical specimens were isolated using collagenase (Sigma‐Aldrich) according to a previously published protocol.^24^ Informed consent was obtained from the patients according to institutional guidelines. The clinicopathological data of the SS patients are shown in Table S1. All cells were cultured in Dulbecco's modified Eagle medium (Sigma‐Aldrich) supplemented with 10% fetal bovine serum (FBS; Sigma‐Aldrich) and penicillin/streptomycin (Sigma‐Aldrich) in a humidified atmosphere of 5% CO_2_ at 37°C. The chemical reagents used in this study are listed in Table S2.

### 2.2 Light irradiation

For the in vitro experiments, Teleopto LED array systems (LEDA‐X LED Array with LAD‐1 driver; Amuza) were used. The cells received continuous light irradiation in a CO_2_ incubator with blue (peak at 470 nm), green (peak at 525 nm), or red (peak at 630 nm) light. For in vivo experiments or the chorioallantoic membrane (CAM) assay, LED chips (WS2812B; WorldSemi) were used at a light intensity of 30 mW/cm^2^, which peak at 470 nm for the output of BL. The light intensity was measured with a Light Power Meter (LPM‐100; Amuza). After BL irradiation, the samples were used for further study. See the Data S1 for further details on the experimental methods.

### 2.3 CAM assay

Fertilized chicken eggs were purchased from the Goto farm in Gifu, Japan. The fertilized chicken eggs were kept in a humidified egg incubator at 37°C. After 11 days of incubation, a window on the eggshell was made. For transplantation, a sterile Teflon ring (Sansyo) was placed at the Y‐shape blood vessel on the CAM. Then, 20 μL of a cellular suspension containing 5 × 10^5^ SYO‐1 cells in growth medium were grafted into the ring and the window was covered with clear film. And 4 days after transplantation, irradiation with BL (30 mW/cm^2^) was started continuously. One week after transplantation, CAM tumors were resected and fixed in 4% paraformaldehyde. Hematoxylin and eosin (H&E) staining and TUNEL analysis were conducted as described in Data S1.

### 2.4 In vivo xenograft tumor model

Male BALB/c nude mice were purchased from SLC Japan at 4 weeks of age. All animal experiments were performed in accordance with the university's guidelines on the ethical care and use of animals. The mice were kept in the laboratory for 1 week to adapt to the rearing environment. Then 100 μL cellular suspension containing 5 × 10^6^ SYO‐1 cells in PBS were injected subcutaneously into the left flank of the mice. One week after injection, the mice were randomly divided into two groups: control group (CTL, *n* = 8) and BL group (BL, *n* = 8). Mice in the BL group were fixed on cages using adhesive tapes and irradiated with BL (30 mW/cm^2^) for 8 h/day for 12 days. Mice in the CTL group were fixed in the same manner without BL irradiation. Body weight was measured every 2 or 3 days, the long and short diameters of the tumor were measured using an electronic caliper, and the tumor volume was calculated. After BL irradiation, the mice were sacrificed. The tumors were extracted, weighed, and used for further study.

### 2.5 Statistical analyses

Plot and statistical tests were generated with GraphPad Prism 9 (GraphPad Software). Data are presented as the mean ± standard error of the mean. All results were confirmed in at least three independent experiments. An unpaired Student's *t*‐test or one‐way analysis of variance was used to calculate the significance between different groups. *p* < 0.05 was considered statistically significant.

## 3 RESULTS

### 3.1 BL inhibits viability and colony formation of SS cells and induces histological changes in the CAM SS tumor model

**FIGURE 1:**
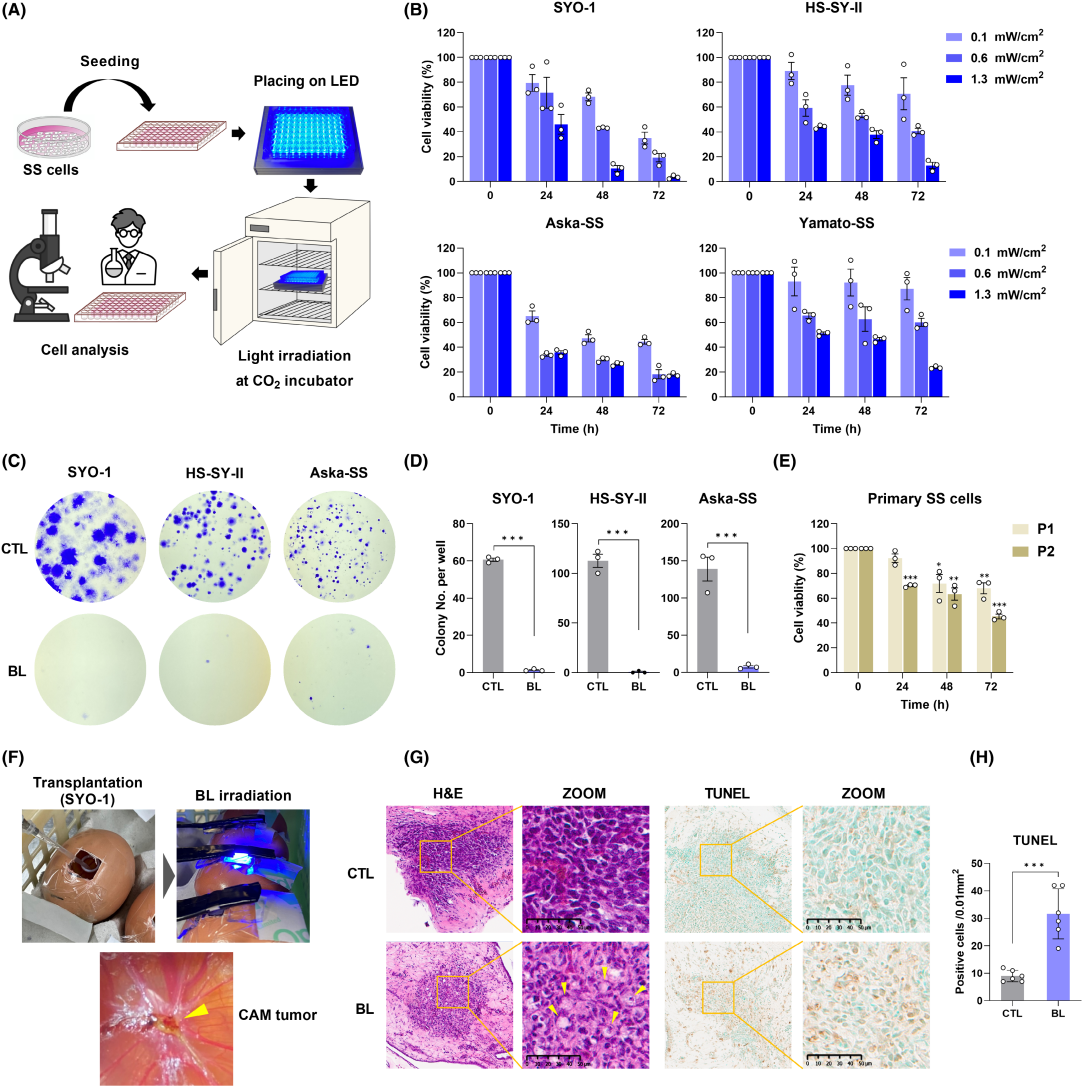
Blue light (BL) inhibited the proliferation and colony formation capacity of synovial sarcoma (SS) cells and induced a histological change in chorioallantoic membrane (CAM) tumor tissues. (A) Schematic illustration of the experimental protocol using LED devices in vitro. (B) A CCK‐8 assay was used to measure cell viability. (C) Colony formation assay of SS cells incubated with or without BL irradiation (0.1 mW/cm^2^) for 24 h. (D) Quantification of the mean number of colonies formed in each group. (E) A CCK‐8 assay was used to measure the cell viability of primary SS cells from two patients with SS (P1 and P2). (F) Images of BL irradiation in the CAM assay. The yellow arrow indicates the formation of a tumor‐like structure. (G) H&E staining was used to evaluate the histology. The yellow arrows indicate vacuolar change in the cytoplasm of tumor cells. The apoptotic status of tumor tissues was assessed by the TUNEL assay. (H) Quantification of TUNEL‐positive cells per field. Data are presented as the mean ± standard error of the mean (SEM) of three independent experiments. ***p < 0.001.

The cells received light irradiation continuously in a CO_2_ incubator and were subjected to biological analyses (Figure 1A). The CCK‐8 assay showed that BL inhibited the viability of SYO‐1, HS‐SY‐II, Saka‐SS, and Yamato‐SS cells in both a light intensity‐ and time‐dependent manner (Figure 1B). In addition, the colony formation ability of SS cells was markedly suppressed by BL (0.1 mW/cm^2^) for 24 h (Figure 1C,D). To determine whether the effect of light irradiation on SS cells varied depending on the wavelength, SS cells were separately irradiated with three different wavelengths of light, blue (peak at 470 nm), green (peak at 525 nm), or red (peak at 630 nm) at a unified intensity of 0.6 mW/cm^2^ for 48 h. As a result, only the inhibition of BL for each cell line was exhibited (Figure S1A). To determine whether BL also has effects on primary human SS cells, primary SS cells isolated from two SS patients (P1 and P2) were irradiated with BL (0.6 mW/cm^2^). As shown in Figure 1E, BL irradiation resulted in the decreased viability of primary SS cells in a time‐dependent manner. The growth‐inhibitory effect of BL on HEK293, a non‐cancer cell line, was also examined, and a growth‐inhibitory effect was observed that was similar to the results for the SS cell lines (Figure S1B).

Next, to verify whether BL has antitumor effects on three‐dimensional structural tissues as tumors in addition to two‐dimensional planar structures such as cultured cells, we performed a CAM assay as a preliminary step before in vivo experiments (Figure 1F). H&E staining of CAM SS tumor tissues irradiated with BL showed decreased cellularity and vacuolar changes in the cytoplasm of tumor cells compared to CTL (Figure 1G), and TUNEL analysis showed a significant increase in the number of TUNEL‐positive cells (Figure 1G,H).

### 3.2 BL suppresses the migration and invasion of SS cells

To investigate the molecular mechanism underlying the growth‐inhibitory effect of BL on SS cells, we performed microarray analysis in which SYO‐1 cells were incubated with or without BL (0.6 mW/cm2) for 48 h. The volcano plot of the differentially expressed genes (DEGs) indicated that there were 1131 DEGs in BL‐irradiated SS cells compared with CTL ones, among which 605 DEGs were significantly upregulated and 526 were significantly downregulated. Differences in expression levels were defined by cutoff log2 (fold change) > 1 and corrected *p* > 0.05 (Figure S2A). Unsupervised hierarchical clustering analysis of these genes revealed a clear separation of DEGs between BL‐irradiated cells and CTL cells (Figure S2B). To further characterize the overarching biological process, gene set enrichment analysis (GSEA) of the hallmark and Gene Ontology gene sets were performed. Table S3 shows the top upregulated or downregulated gene sets based on a normalized enrichment score (NES) of >1.5 or <−1.5 and a normal *p*‐value of <0.05 as the threshold.

**FIGURE 2:**
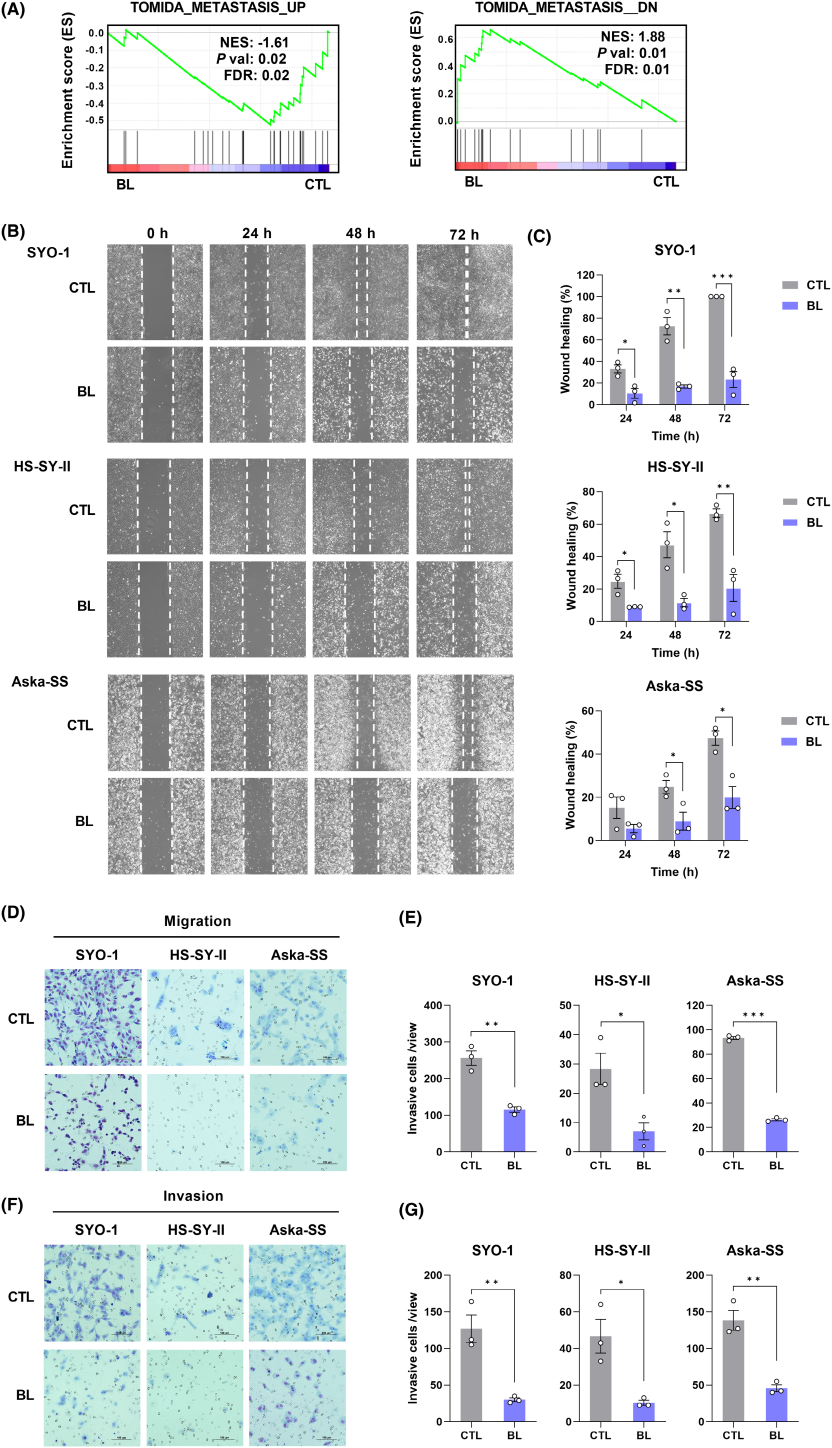
Blue light (BL) suppressed the migration and invasion abilities of synovial sarcoma (SS) cells. (A) gene set enrichment analysis (GSEA) of microarray data. NES, normalized enrichment score; FDR, false discovery rate. (B) Wound healing assay of SS cells. (C) Quantification of the mean percentage of wound healing in each group. (D) The migration abilities were analyzed using Transwell filters. (E) Quantification of the mean number of migrated cells in each group. (F) Invasion ability was analyzed using Matrigel‐coated Transwell filters. (G) Quantification of the mean number of invasive cells in each group. Data are presented as the mean ± SEM of three independent experiments. *p < 0.05, **p < 0.01, ***p < 0.001.

The results of GSEA showed a negative correlation for metastasis_up and a positive correlation for metastasis_down, indicating that BL inhibits metastasis of SS cells (Figure 2A). To verify the inhibitory effect of BL on the migration ability of SS cells, we performed a wound healing assay, which showed that BL (0.6 mW/cm^2^) reduced the migration ability of SYO‐1, HS‐SY‐II, and Aska‐SS cells (Figure 2B,C). Furthermore, we performed Transwell experiments and found that the migration and invasion abilities of SS cells were much lower in BL‐irradiated cells compared with CTL cells (Figure 2D–G).

### 3.3 BL induces apoptosis in SS cells

**FIGURE 3:**
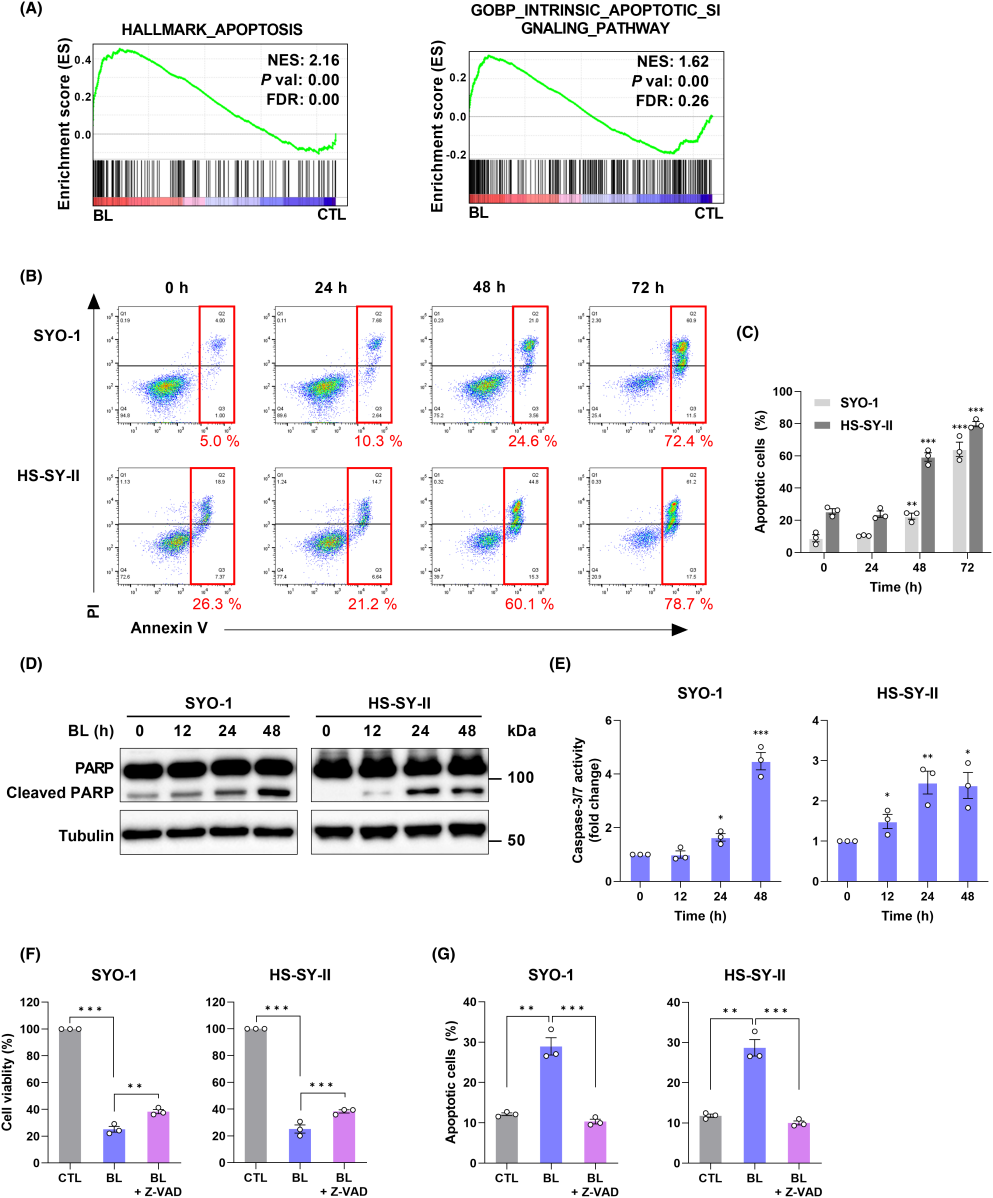
Blue light (BL) induced apoptosis in synovial sarcoma (SS) cells. (A) Gene set enrichment analysis (GSEA) of microarray data. NES, normalized enrichment score; FDR, false discovery rate. (B, C) The ratio of apoptotic cells was measured and analyzed by Annexin V‐FITC/PI staining and flow cytometry (FC). (D) Caspase‐3/7 activity was measured by CellEvent staining and FC. (E) Western blot analysis of PARP and cleaved PARP. (F) The CCK‐8 assay was used to measure the cell viability of BL‐irradiated SS cells in the presence or absence of a pan‐caspase inhibitor, Z‐VAD‐FMK (Z‐VAD, 100 μM). (G) The ratio of apoptotic cells was measured and analyzed by Annexin V‐FITC/PI staining and FC. Data are presented as the mean ± SEM of three independent experiments. *p < 0.05, **p < 0.01, ***p < 0.001. The quantification of western blot bands is presented in Figure S7A.

Among related gene sets in GSEA, apoptosis‐related signatures were enriched (Figure 3A); therefore, we first investigated whether BL promoted apoptosis in SS cells. Annexin V‐FITC/PI double staining with flow cytometry showed that BL (0.6 mW/cm^2^) significantly increased the proportion of apoptotic SYO‐1 and HS‐SY‐II cells in a time‐dependent manner (Figure 3B,C). Next, cell cycle analysis was performed to determine whether inhibition of cell proliferation was caused by cell cycle arrest. To clarify the protein underlying BL‐induced apoptosis, we showed that BL (0.6 mW/cm^2^) markedly induced the expression of cleaved PARP in SYO‐1 and HS‐SY‐II cells (Figure 3D). To determine whether caspase activation was directly involved in BL‐induced apoptotic events, CellEvent fluorogenic substrate was utilized via flow cytometry. Caspase 3/7 activation was detectable in BL (0.6 mW/cm^2^)‐irradiated SYO‐1 and HS‐SY‐II cells in a time‐dependent manner (Figure 3E). Additionally, to better characterize whether BL‐induced cell death was caspase‐dependent, the cells were irradiated with BL in the presence or absence of the pan‐caspase inhibitor, Z‐VAD‐FMK (Z‐VAD). BL (0.6 mW/cm^2^) with Z‐VAD (100 μM) for 48 h sufficiently rescued cell viability (Figure 3F) and decreased the number of apoptotic cells, according to an Annexin V‐FITC/PI assay of SYO‐1 and HS‐SY‐II cells (Figure 3G,H). These findings suggest that BL induces apoptosis by caspase activation in SS cells.

### 3.4 ROS are critical for the BL‐induced apoptosis of SS cells

**FIGURE 4:**
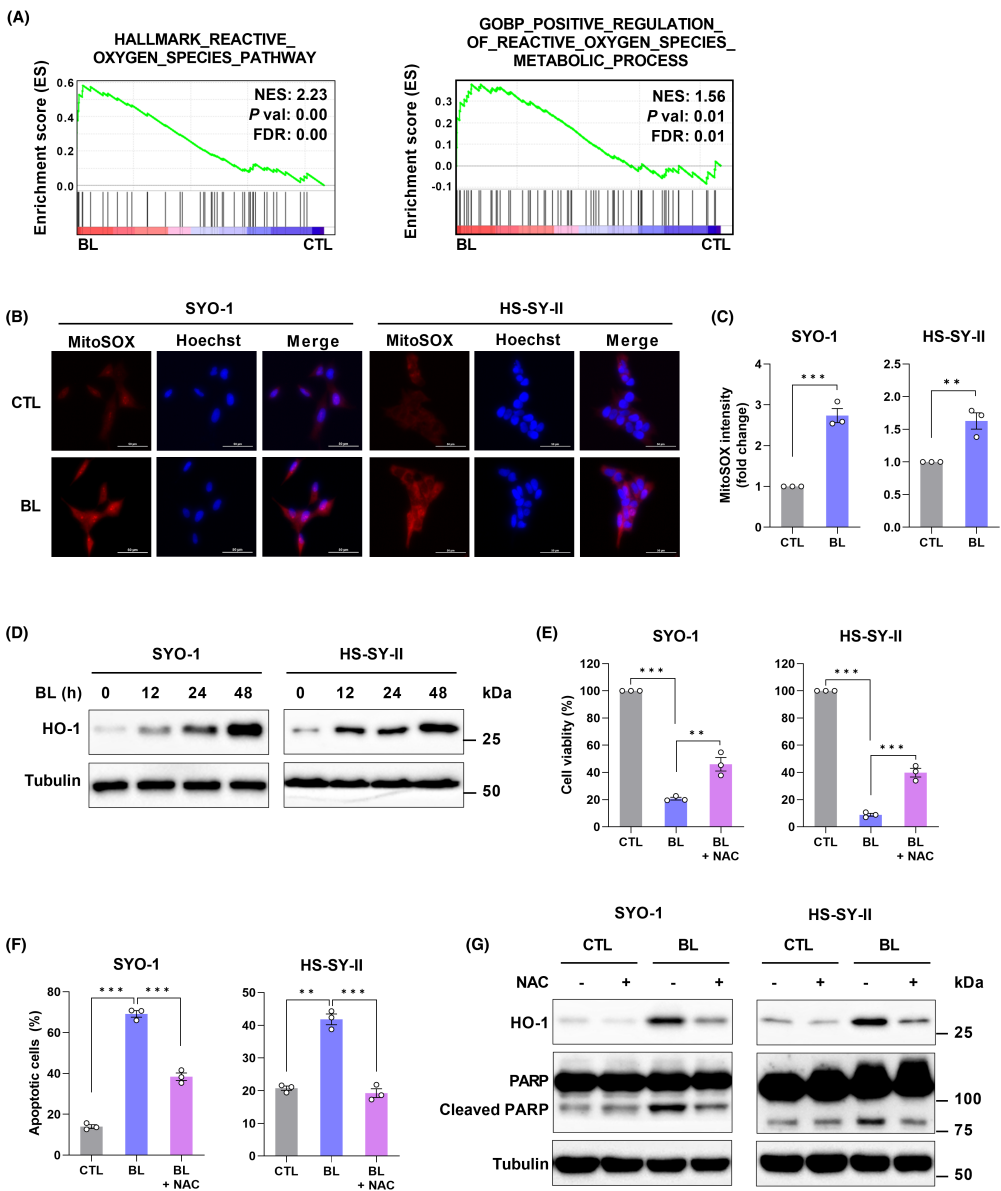
ROS are critical in BL‐induced apoptosis in synovial sarcoma (SS) cells. (A) GSEA of microarray data. NES, normalized enrichment score; FDR, false discovery rate. (B) The production of mitochondrial ROS was assessed using a MitoSOX assay. Representative images of MitoSOX‐stained cells captured by fluorescent microscopy are shown. (C) Quantification of mitochondrial ROS (MitoSOX) was measured by flow cytometry (FC). (D) The protein level of HO‐1 was determined by western blotting. (E) The CCK‐8 assay was used to measure the cell viability of BL‐irradiated SS cells in the presence or absence of the ROS scavenger, N‐acetyl‐cysteine (NAC, 5 mM). (F) The ratio of apoptotic cells was measured and analyzed by Annexin V‐FITC/PI staining and FC. (G) Western blot analysis of HO‐1, PARP, and cleaved PARP. Data are presented as the mean ± SEM of three independent experiments. *p < 0.05, **p < 0.01, ***p < 0.001. The quantification of western blot bands is presented in Figure S7B–D.

GSEA of microarray data showed that ROS‐related signatures were enriched (Figure 4A). To verify ROS production in BL‐irradiated SYO‐1 cells, the CellROX probe was initially used. As shown in Figure S3A,B, irradiation with BL (0.6 mW/cm^2^) for 48 h increased the percentage of cells with green fluorescence. The major source of ROS in cells is mitochondria^25^; therefore, we next investigated whether ROS formation linked to BL irradiation occurred in the mitochondria. We used MitoSOX, a probe that specifically detects ROS produced in mitochondria. As expected, BL irradiation caused enhanced ROS production in the mitochondria (Figure 4B,C).

GSEA analysis of microarray data also showed gene enrichment in the cellular oxidant detoxification and antioxidant activity in BL‐irradiated SYO‐1 cells (Figure S3C). Overproduction of ROS caused by BL was expected to result in oxidative stress as well as induce cytoprotective and antioxidant activity in SS cells. In the analysis of gene expression of HO‐1,^26^ oxidative stress‐induced growth inhibitor 1 (OSGIN1),^27^ and NAD(P)H:quinone oxidoreductase 1 (NQO1),^28^ which are related to oxidative stress, the mRNA levels of BL‐irradiated SS cells were higher than those of CTL cells (Figure S3D). Western blot analysis showed that SYO‐1 and HS‐SY‐II cells irradiated with BL (0.6 mW/cm^2^) sharply increased protein expression of HO‐1 (Figure 4D), which is a rate‐limiting enzyme in heme catabolism and plays a key role in inducible antioxidant defenses.^29^, ^30^ These results revealed that BL increased the production of mitochondrial ROS, causing oxidative stress in SS cells.

In addition, considering that overaccumulation of ROS might cause the apoptotic death of SS cells, the experiments were performed using BL irradiation with or without N‐acetyl‐cysteine (NAC), a ROS scavenger. NAC (5 mM) significantly reversed the inhibition of cell viability induced by BL (0.6 mW/cm^2^) for 48 h (Figure 4E) and decreased the number of apoptotic cells in the Annexin V‐FITC/PI assay (Figure 4F,G). The protein expression of HO‐1 and cleaved PARP was downregulated in SYO‐1 and HS‐SY‐II cells irradiated with BL (0.6 mW/cm^2^) combined with NAC (5 mM) for 48 h compared with cells irradiated with BL alone (Figure 4H). These results demonstrated that mitochondrial ROS mediates BL‐induced apoptosis in SS cell lines.

### 3.5 BL triggers the mitochondrial dysfunction caused by ROS production

**FIGURE 5:**
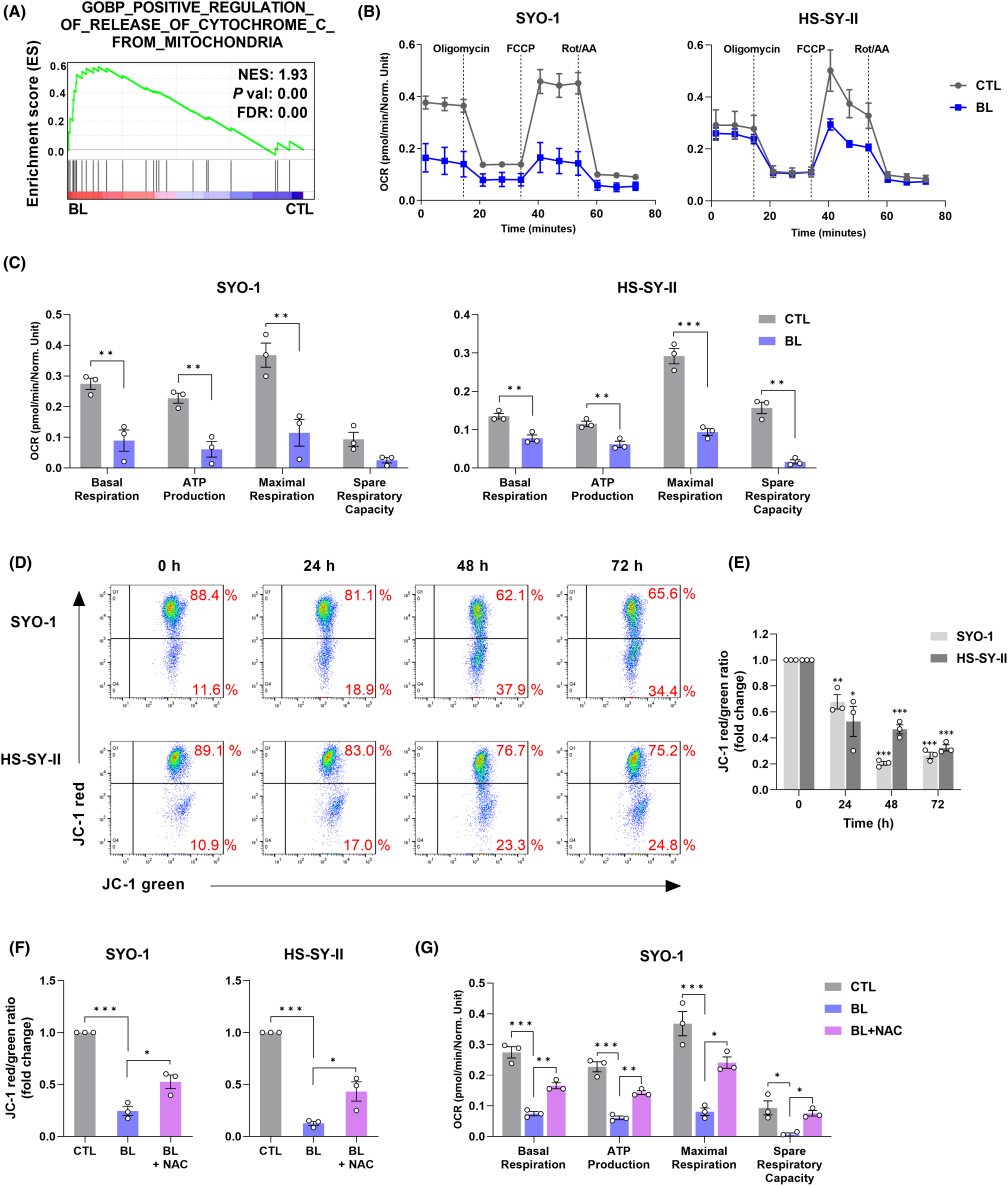
Blue light (BL) induced ROS production, thereby triggering mitochondrial dysfunction. (A) GSEA of microarray data. NES, normalized enrichment score; FDR, false discovery rate. (B) The OCR was measured with the Seahorse XF Cell Mito Stress assay using the Seahorse XF HS Mini analyzer. (C) Quantification of metabolic parameters. (D, E) The change in mitochondrial membrane potential (MMP) was measured by JC‐1 and flow cytometry. (F) The MMP of BL‐irradiated SS cells was measured by JC‐1 in the presence or absence of NAC (5 mM). The data are presented as the mean ± SEM of three independent experiments. *p < 0.05, **p < 0.01, ***p < 0.001.

GSEA of microarray data indicated the positive regulation of cytochrome c release from mitochondria in BL‐irradiated cells (Figure 5A). Some sort of stress signal may trigger mitochondrial dysfunction, resulting in the release of cytochrome c and thereby causing caspase activation and apoptosis.^31^ Next, we investigated whether BL might amplify mitochondrial dysfunction in SS cells. Because the mitochondrial oxidative phosphorylation process may be affected by mitochondrial dysfunction, we first investigated the mitochondrial respiratory capacity, using the Seahorse XF HS analyzer. The OCR, an indicator of mitochondrial respiration, showed that BL (0.6 mW/cm^2^) for 48 h remarkably suppressed basal respiration, ATP production, maximal respiration, and spare respiratory capacity in SYO‐1 and HS‐SY‐II cells (Figure 5B,C). Then the change in mitochondrial membrane potential (MMP), an important factor in mitochondrial dysfunction, was measured with JC‐1 staining by flow cytometry. When mitochondria are healthy, JC‐1 forms aggregates and emits red fluorescence, but when the MMP is reduced, JC‐1 becomes monomeric and emits green fluorescence. Therefore, the ratio of red to green fluorescence is used as an indicator of mitochondrial damage. We observed a time‐dependent decrease in the ratio of red/green fluorescence induced by BL (0.6 mW/cm^2^) (Figure 5D,E).These results indicated that BL induced severe damage in the mitochondria of SS cells.

Increased ROS might interfere with the MMP and electron transport chain for ATP synthase, upsetting the balance of energy homeostasis and causing cell death.^32^, ^33^ Hence, to further analyze the link between ROS and mitochondrial dysfunction, SS cells were irradiated with BL (0.6 mW/cm^2^) with or without NAC (5 mM) for 48 h. Notably, the loss of MMP was remarkably reversed in the presence of NAC (Figure 5F,G). These results demonstrate that ROS generated by BL were responsible for the mitochondrial dysfunction.

### 3.6 Autophagy occurs in response to BL‐induced ROS and promotes SS cell survival

**FIGURE 6:**
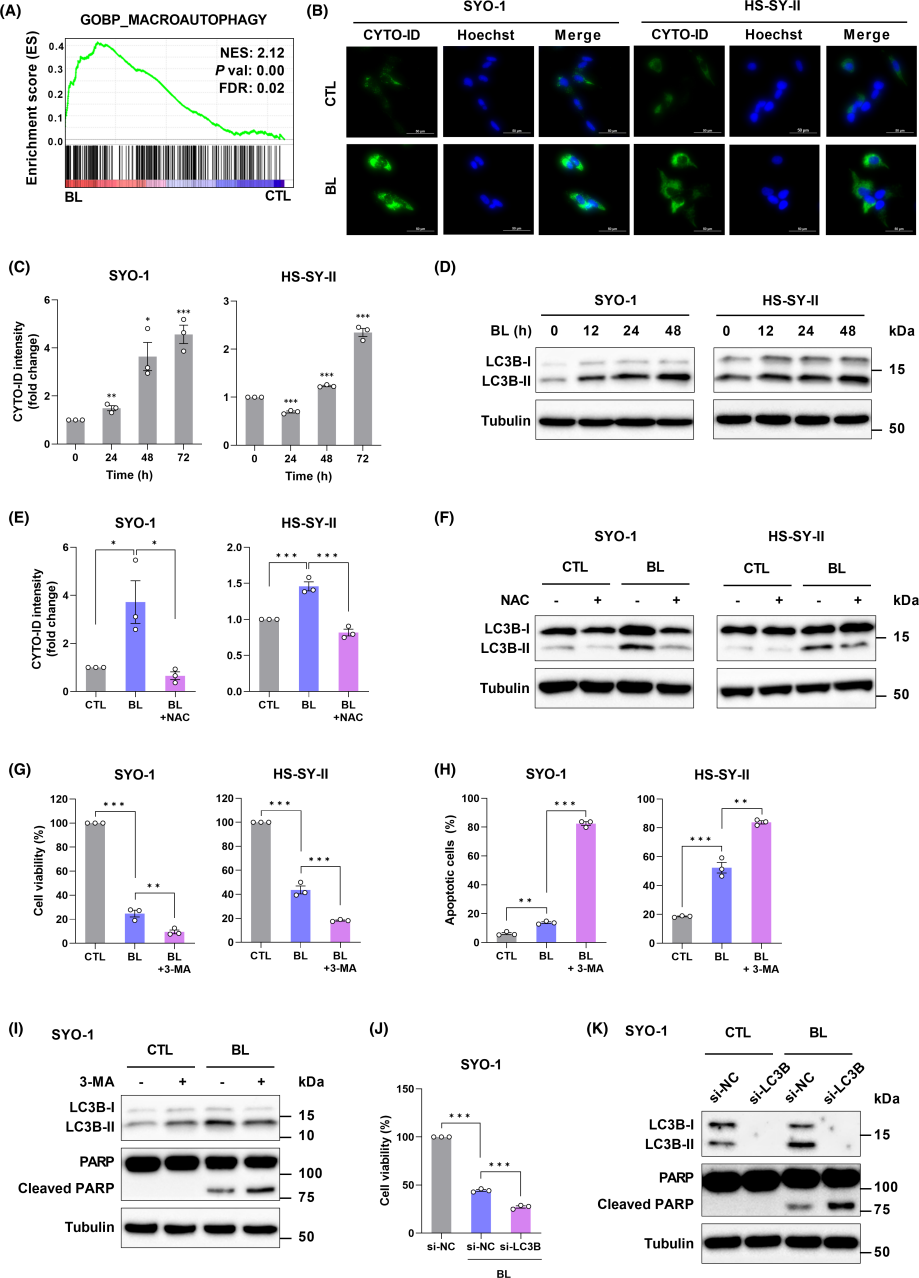
Autophagy occurs in response to Blue light (BL)‐induced ROS and promotes synovial sarcoma (SS) cell survival. (A) GSEA of microarray data. FDR, false discovery rate. (B) The formation of autophagosomes in SS cells was measured by CYTO‐ID staining. Representative images of CYTO‐ID‐stained cells captured by fluorescent microscopy are shown. (C) Quantification of intracellular autophagosome (CYTO‐ID) was measured by flow cytometry (FC). (D) Western blot analysis of LC3B‐I and LC3B‐II. (E) The formation of autophagosomes in BL‐irradiated SS cells was assessed by CYTO‐ID in the presence or absence of NAC (5 mM). (F) Western blot analysis of LC3B‐I and LC3B‐II. (G) The cell viability of BL‐irradiated SS cells was assessed by the CCK‐8 assay in the presence or absence of the autophagy inhibitor, 3‐MA (5 mM). (H) The ratio of apoptotic cells was measured by Annexin V‐FITC/PI staining and FC. Representative dot plot data are presented. (I) Western blot analysis of LC3B‐I, LC3B‐II, PARP, and cleaved PARP. (J) The cell viability of SYO‐1 cells transfected with LC3B siRNA was measured by the CCK‐8 assay. (K) Western blot analysis of LC3B‐I, LC3B‐II, PARP, and cleaved PARP. Data are presented as the mean ± SEM of three independent experiments. *p < 0.05, **p < 0.01, ***p < 0.001. The quantification of western blot bands is presented in Figure S7E–H.

Autophagy‐related signatures were enriched (Figure 6A); therefore, we determined whether autophagy was activated by BL. The CYTO‐ID fluorescent probe specifically labeling autophagosomes in live cells was used. The results revealed that BL irradiation (0.6 mW/cm^2^) for 48 h stimulated the formation of autophagosomes in SYO‐1 and HS‐SY‐II cells (Figure 6B), and flow cytometry analysis further confirmed these time‐dependent findings (Figure 6C). Furthermore, the conversion of LC3‐I to LC3‐II, which are the major molecular players in autophagy signaling, was measured. As shown in Figure 6D, BL (0.6 mW/cm^2^) increased the accumulation of LC3B‐II in SYO‐1 and HS‐SY‐II cells. These results indicated that BL induced autophagy in SS cells.

ROS is well‐known as a signaling molecule in the regulation of autophagy^34^; therefore, the present study assessed whether ROS participated in BL‐induced autophagy in SS cells. Predictably, inhibition of ROS by NAC (5 mM) attenuated the formation of autophagosomes (Figure 6E) and the expression of LC3B‐II in SS cells (Figure 6F) induced by BL, suggesting that BL‐induced autophagy was mediated by ROS accumulation in SS cells. The close and complex interplay between apoptosis and autophagy has been demonstrated by the results of numerous evidence‐based studies.^35^ To clarify the interplay between autophagy and apoptosis in SS cells, we used 3‐meghylademine (3‐MA) to inhibit autophagy under BL irradiation. The CCK‐8 and Annexin V‐FITC/PI assays showed that BL in combination with 3‐MA (5 mM) remarkably enhanced the effects of BL on cell viability (Figure S4) and apoptotic cell death (Figure 6G,H). We confirmed the results using western blotting and showed that SYO‐1 cells irradiated with BL in the presence of 3‐MA increased the levels of cleaved PARP (Figure 6I). To further confirm the connection between autophagy and apoptosis, specific siRNA against LC3B was applied. Knockdown of LC3B promoted inhibition of cell growth (Figure 6J) and the expression of cleaved PARP (Figure 6K). Taken together, BL induced autophagy, which promoted the survival of SS cells.

### 3.7 BL suppresses the growth of SS cells in vivo

**FIGURE 7:**
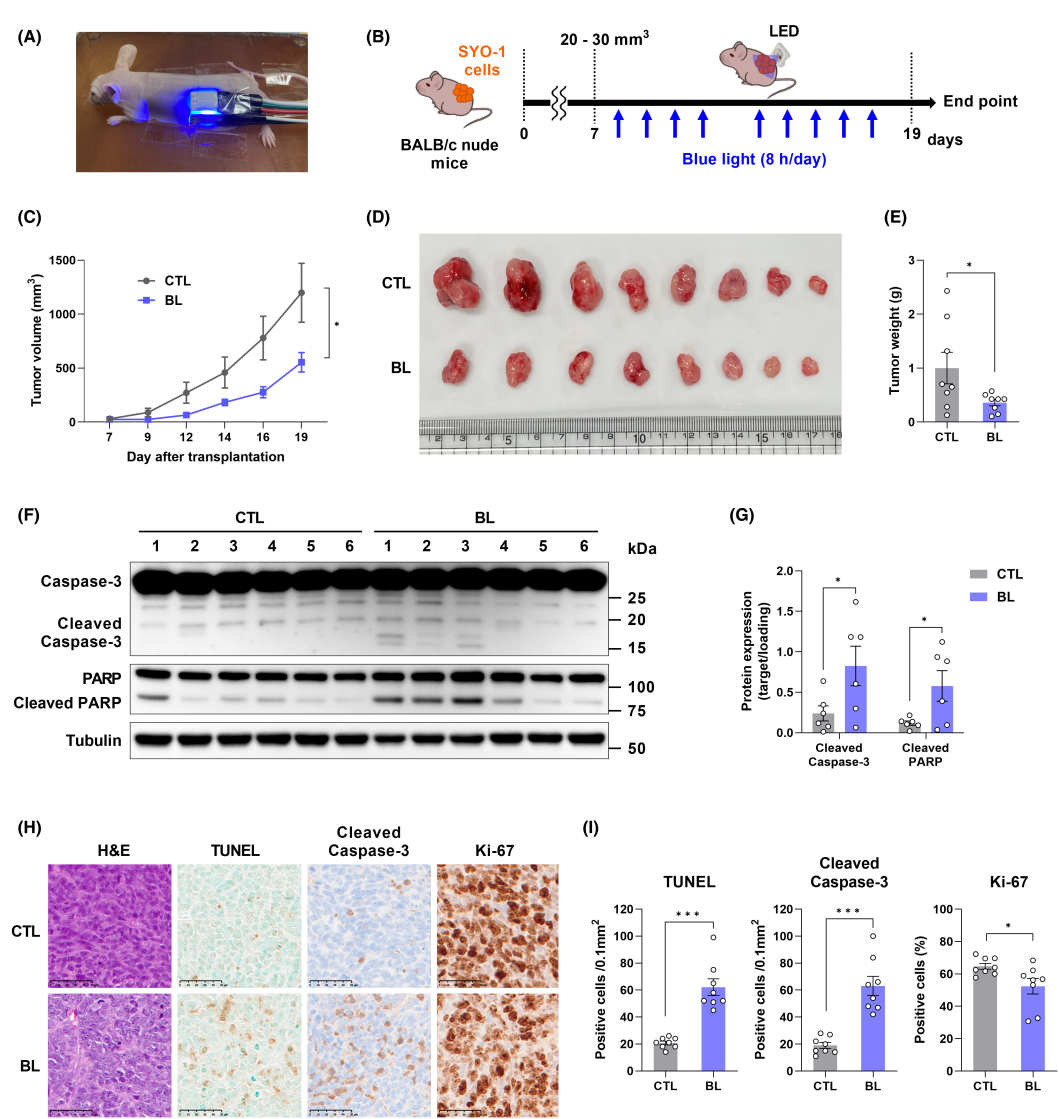
Blue light (BL) suppresses the growth of synovial sarcoma (SS) in vivo. (A) Image of BL irradiation in SYO‐1 tumor‐bearing nude mice. (B) Schematic illustration of the experimental protocol in vivo. (C) Tumor volumes in BALB/c nude mice during the 12‐day BL irradiation. (D) Images of SYO‐1 cells xenograft tumors at the end of the BL irradiation. (E) The weight of SYO‐1 cell xenograft tumor at the end of the treatment. (F) Western blot analysis of the resected xenograft tumor. (G) Quantification of the protein expression is presented. (H) H&E staining was used to evaluate the histology. The apoptotic status of tumor tissues was assessed by the TUNEL assay. The expression of cleaved caspase‐3 and Ki‐67 was also examined by immunohistochemistry. (I) Quantification of TUNEL‐ and cleaved caspase‐3‐positive cells per field, and the percentage of Ki‐67‐positive cells. Data are presented as the mean ± SEM. *p < 0.05, ***p < 0.001.

Finally, we investigated the effect of BL on the in vivo growth of SS cells by subcutaneously injecting SYO‐1 cells into BALB/c nude mice. One week after injection, the mice were irradiated with BL (30 mW/cm^2^, 8 h/day) for 12 days (Figure 7A,B). BL significantly inhibited tumor growth (Figure 7C), whereas the body weights of the mice in the CTL or BL group remained equal (Figure S5). The excised tumors showed that BL‐induced tumors were much smaller than those of the CTL group (Figure 7D). The average weight of tumors in the BL group (360 ± 60 mg) was significantly lower than that of the CTL group (1002 ± 292 mg) (Figure 7E). After the mice were sacrificed, tumors were removed and Western blotting was performed. The results showed that the expression of apoptosis‐related proteins (cleaved PARP and cleaved caspase‐3) was increased in the BL group (Figure 7F,G), which is inconsistent with the in vitro findings. Moreover, BL‐irradiated tumor tissues showed significant increases in TUNEL‐positive cells and cleaved caspase‐3 as well as a decrease in Ki‐67‐positive cells (Figure 7H,I). In addition, H&E staining of the skin above the tumor was performed to investigate the effect of BL on the skin tissue at the site of BL irradiation. No obvious skin damage due to BL irradiation was observed (Figure S6). These data suggest that BL exhibited a potent antitumor effect on SS in vivo that is safe with no side effects on normal tissues.

## 4 DISCUSSION

In this study, we demonstrated that BL has a growth‐inhibitory effect on SS cells and inhibits their migration and invasive ability. BL irradiation led to apoptosis through ROS‐induced mitochondrial dysfunction, which caused the release of cytochrome c from mitochondria and induced apoptosis via the caspase pathway. Moreover, mitochondrial ROS accumulation induced autophagy, thereby inhibiting apoptosis and promoting cell survival. In addition, the inhibitory effect and safety of BL on SYO‐1 xenograft models were demonstrated.

Mitochondria are said to contain chromophores such as cytochrome oxidase or flavin that absorb BL in the wavelength range of around 400–500 nm.^36^, ^37^, ^38^ When exposed to BL, chromophores become excited and react with intracellular oxygen molecules to generate singlet oxygen, which is one of the main ROS and is thought to cause mitochondrial dysfunction and damage to the respiratory chain complex. Excessive generation of mitochondrial ROS can lead to cell death. The fact that a growth‐inhibitory effect on SS cells was observed only with BL and not with green and red light (Figure S1A) is considered to be due to the particular reaction of chromophores in mitochondria to BL.

The advantage of using BL as described in this paper is that it is less invasive than surgical treatment and is expected to cause less damage to the skin and other peri‐tumor tissue compared with radiation therapy, because its wavelength is the visible light spectrum. Also, compared with chemotherapy, there are no systemic side effects. However, a possible adverse effect of BL on normal tissues is the generation of cytotoxic ROS in normal cells as well as SS cells, which causes oxidative damage to the cells. Nevertheless, it has been reported that cancer cells have higher ROS levels compared with normal cells and are able to delicately balance ROS with antioxidants in order to maintain their carcinogenic potential.^39^ That is to say, cancer cells and normal cells differ in their cellular response to ROS, and cancer cells may be more inclined toward apoptosis resulting from an imbalance in the intracellular antioxidant system caused by excessive production of ROS, suggesting that BL might be applied to selectively target cancer cells. In this study, BL did not affect mouse skin tissue (Figure S6) but did have a growth‐inhibitory effect on HEK293 (Figure S1B), a non‐cancer cell line. This in vitro result does not necessarily reflect the adverse effects of BL on normal tissues because the cell line is an immortalized cell that has undergone an extreme increase in proliferative capacity due to transformation and is not, strictly speaking, a normal cell.

This study identified autophagy inhibitors as candidate agents that enhance the anti‐tumor effects of BL on SS. In cancer therapy research, autophagy has been reported to act on either cell survival or cell death.^40^ Autophagic cell death has been reported in previous in vitro studies on the antitumor effects of BL on colon cancer cells^14^ and osteosarcoma cells.^18^ In the present study, we demonstrated that, in contrast to those reports, BL‐induced autophagy is responsible for cell survival in SS (Figure 6G–K). Furthermore, we showed that 3‐MA, an autophagy inhibitor, enhances the BL‐induced apoptosis in SS cells (Figure 6G–I). These results suggest that autophagy inhibitors such as 3‐MA might be used as sensitizers of BL. However, given that we have only been able to verify this in vitro, future studies on the in vivo antitumor‐enhancing effects of autophagy inhibitors on BL are needed.

We also found that continuous irradiation of SS cells with a very low‐power BL source showed marked antitumor effects. All previous studies that we have been able to find used high‐power BL irradiation for 30 min to 24 h to investigate the effects on cancer cells.^9^, ^10^, ^11^, ^12^, ^13^, ^14^, ^15^, ^16^, ^17^, ^18^, ^19^, ^20^, ^21^, ^22^ The concept of our study, with an eye toward initial clinical application, was to implant small wireless LEDs in the body and continuously irradiate the tumor. Therefore, it was necessary to develop an experimental model in which BL was irradiated at the lowest possible power for a long period of time, for example, up to 72 h. Recent studies have reported remarkable progress in the development of small wireless LEDs, and data from mouse animal experiments using small wireless LEDs in the fields of photodynamic therapy^41^ and photoimmunotherapy^42^ suggest the potential for these devices in BL therapy applications. If treatment involving BL irradiation of soft‐tissue sarcomas using small wireless LEDs becomes feasible, it would provide a minimally invasive local adjuvant therapy with fewer side effects compared with conventional chemotherapy or radiation therapy.

We consider there to be two main limitations of this study. First, the in vivo studies did not confirm the occurrence of BL‐induced autophagy in SS, which was demonstrated in the in vitro studies. It is therefore necessary to confirm the protein expression levels of LC3 by Western blotting or tissue immunostaining, as was performed in the verification of apoptosis. We have not yet been able to verify autophagy because we could not obtain sufficient amounts of tumor proteins or suitable antibodies for tissue immunostaining. We first need to verify BL‐induced autophagy in vivo in order to use autophagy inhibitors as sensitizers for BL, as described above. Second, we have not yet been able to examine the depth to which BL penetrates and exerts its antitumor effects on the CAM tumors and mouse‐transplanted tumors used in this study. As is generally known, BL has a shorter wavelength compared with red and near‐infrared light and thus has lower tissue permeability.^43^ If a tumor is larger than those transplanted in the mice in this study, BL might not be effective in shrinking the tumor due to its poor penetrability. We therefore need to conduct further experiments using rats or pigs to create larger tumors, investigate the permeability of BL in the tissues of these tumors, and further examine the potential clinical application of BL.

In conclusion, we analyzed in detail the antitumor effects of low‐power continuous irradiation of BL on SS in vitro and in vivo. The results suggest the potential for BL irradiation to be applied in novel, minimally invasive therapies for the treatment of soft tissue sarcomas, including SS. In addition, with the advancement of LED technology, it may be possible to overcome the poor tissue permeability of BL by placing the light source near the tumor and performing continuous low‐power irradiation that does not damage normal tissue as well as to enhance the effect of BL via combination with an autophagy inhibitor.

## AUTHOR CONTRIBUTIONS

**Makoto Takeuchi:** Conceptualization (lead); data curation (lead); formal analysis (lead); investigation (lead); methodology (lead); software (lead); validation (lead); writing – original draft (lead). **Toshihiko Nishisho:** Conceptualization (equal); formal analysis (equal); project administration (lead); writing – review and editing (lead). **Shunichi Toki:** Data curation (equal); project administration (equal); visualization (equal); writing – review and editing (equal). **Shinji Kawaguchi:** Data curation (equal); investigation (equal); visualization (equal). **Shunsuke Tamaki:** Data curation (equal); investigation (equal). **Takeshi Oya:** Conceptualization (equal); data curation (equal); investigation (equal); supervision (equal); writing – review and editing (equal). **Yoshihiro Uto:** Formal analysis (supporting); methodology (equal); writing – review and editing (supporting). **Toyomasa Katagiri:** Supervision (supporting); writing – review and editing (supporting). **Koichi Sairyo:** Funding acquisition (lead); project administration (equal); resources (lead); supervision (lead).

## CONFLICT OF INTEREST STATEMENT

The authors have no conflict of interest.

## ETHICAL APPROVAL STATEMENT

Patients with synovial sarcoma provided informed consent before surgery for a portion of their resection specimens to be used for research. All experiments involving patient samples were performed after obtaining approval from the ethics committee of Tokushima University Hospital (Approval No. 1992–8). All animal experiments were conducted with approval from the Ethics Committee of Tokushima University for Animal Research (Approval No. T2021‐64).

## Supporting information


Data S1

## Data Availability

The data used in this study are available from the corresponding author upon reasonable request.
