# Supplementary material for: Blue light induces apoptosis and autophagy by promoting ROS‐mediated mitochondrial dysfunction in synovial sarcoma
Source: Cancer Med. 2023 Feb 1;12(8):9668–83. doi: 10.1002/cam4.5664 (PMC10166932; doi:10.1002/cam4.5664)
Supplement: Supplementary file 1 — Data S1 [file CAM4-12-9668-s001.zip › cam45664-sup-0001-Figures.pdf]

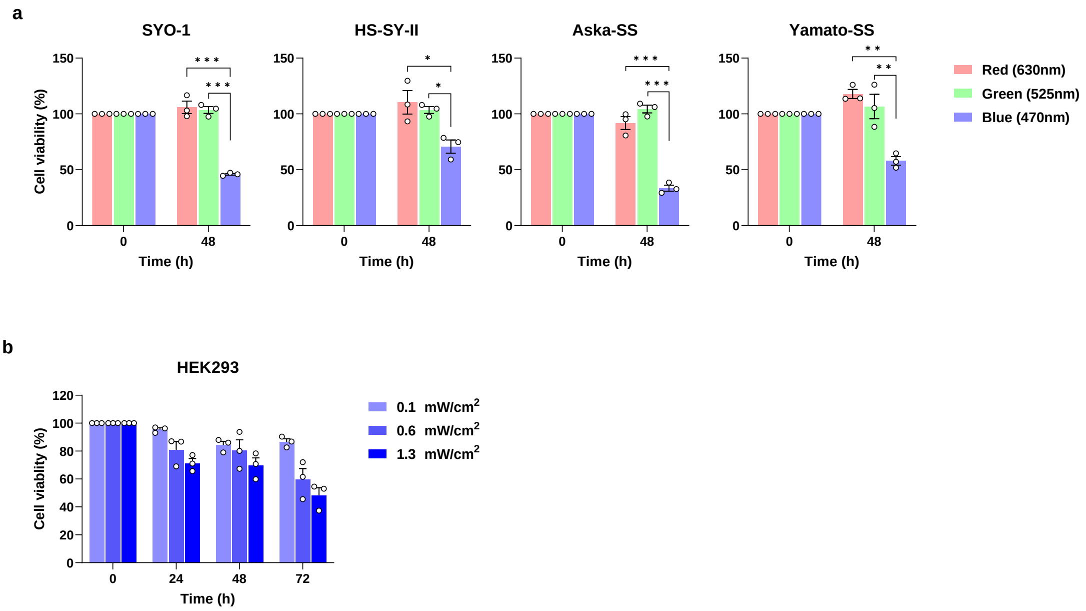

Supplementary Figure 1. (a) SS cells were irradiated with blue (peak at 470 nm), green (peak at 525 nm), or red (peak at 630 nm) light at a light intensity of 0.6 mW/cm^2 for 48 h, after which cell viability was measured with the CCK-8 assay. (b) Results of the CCK-8 assay. Data are presented as the mean ± SEM of three independent experiments. *P < 0.05, **P < 0.01, ***P < 0.001.

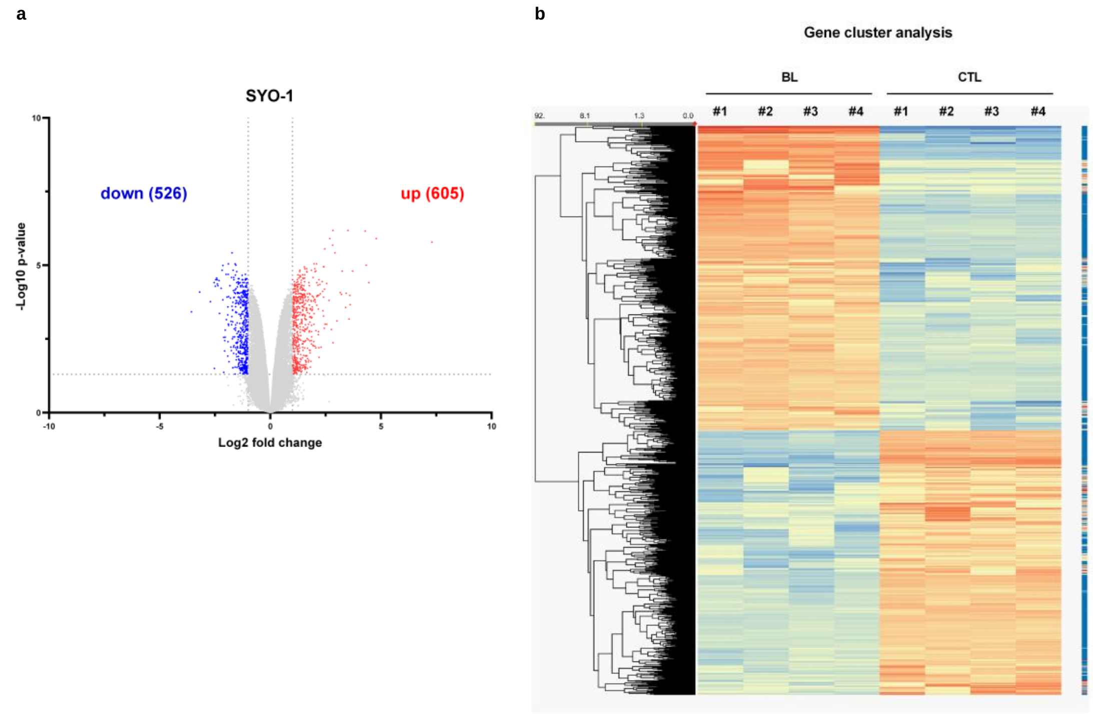

Supplementary Figure 2. (a) Volcano plot visualizing DEGs between the BL group, which was irradiated with BL (0.6 mW/cm^2) for 48 h, and the control group in SYO-1 cells. Red dots (upregulated) and blue dots (downregulated) were defined as DEGs by cutoff log2 (fold change) >1 and corrected *P* > 0.05, and gray dots indicate transcripts that did not change significantly between the two groups. (b) Gene cluster analysis (Euclidean distance, Ward-linked) of 1,131 DEGs with quadruplicate samples was conducted. The X-axis represents the DEGs and the Y-axis represents the samples.

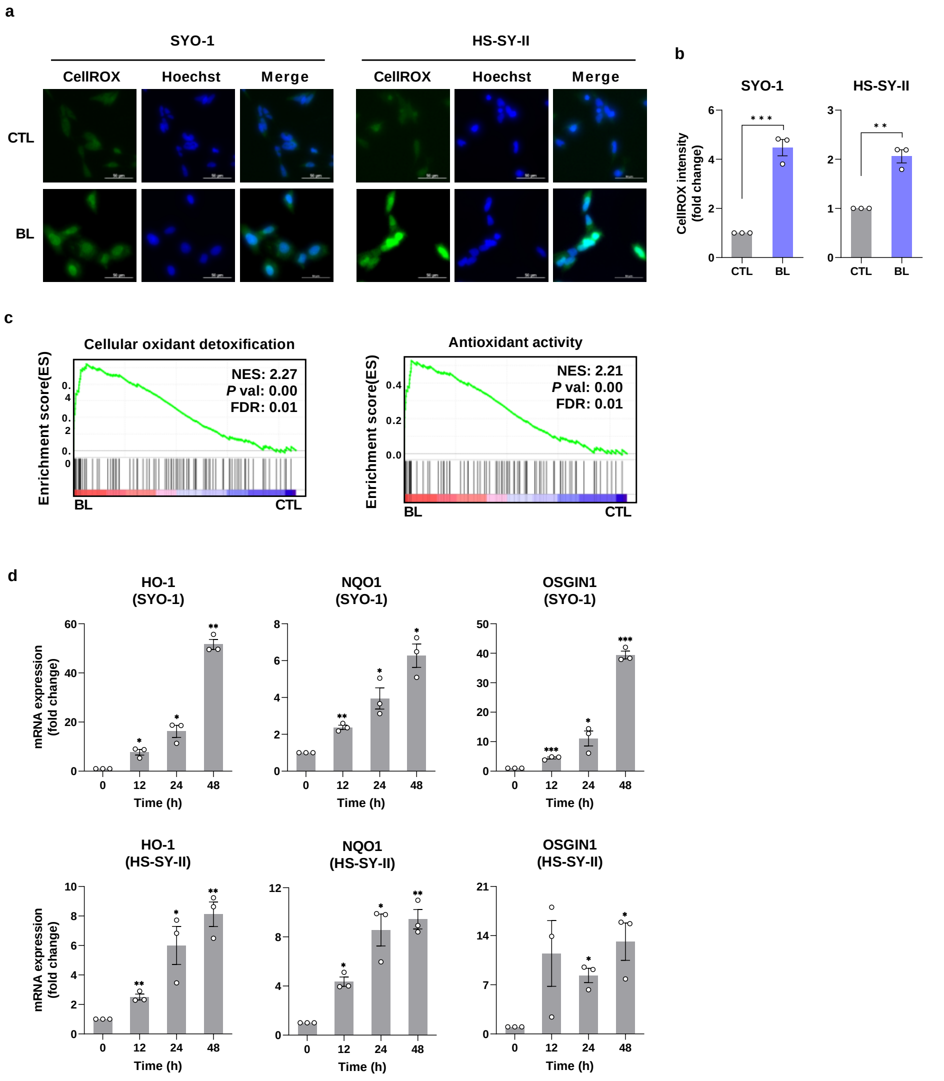

Supplementary Figure 3. (a) The production of intracellular ROS was assessed by the CellROX assay. Representative images of CellROX stained cells captured by fluorescent microscopy are shown. (b) Quantification of intracellular ROS (CellROX) was measured by flow cytometry. (c) GSEA of microarray data. NES: normalized enrichment score; FDR: false discovery rate. (d) The mRNA expression of HO-1, NAD(P)H:quinone oxidoreductase 1 (NQO1), and oxidative stress induced growth inhibitor 1 (OSGIN1) was measured by qPCR. Data are presented as the mean ± SEM of three independent experiments. $^{**}P < 0.01$, $^{***}P < 0.001$.

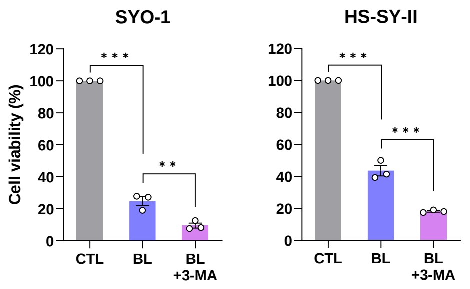

Supplementary Figure 4. SYO-1 and HS-SY-II cells were irradiated with BL (0.6 mW/cm^2) for 48 h in the presence or absence of the autophagy inhibitor 3-MA (5 mM), after which cell viability was assessed by the CCK-8 assay.

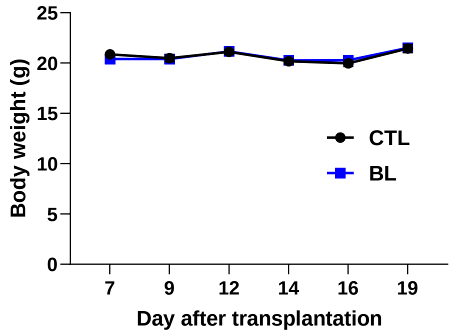

Supplementary Figure 5. Mice body weight were recorded and compared. Data are presented as the mean ± SEM.

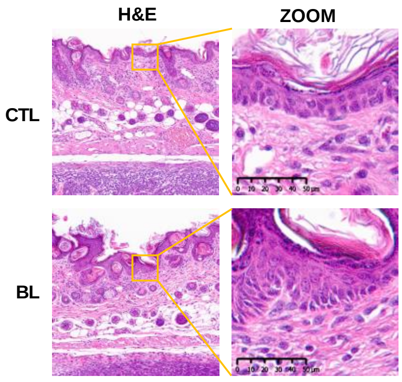

Supplementary Figure 6. H&E staining of mouse skin tissue directly above the transplanted tumor was performed to investigate the effect of BL on the skin tissue.

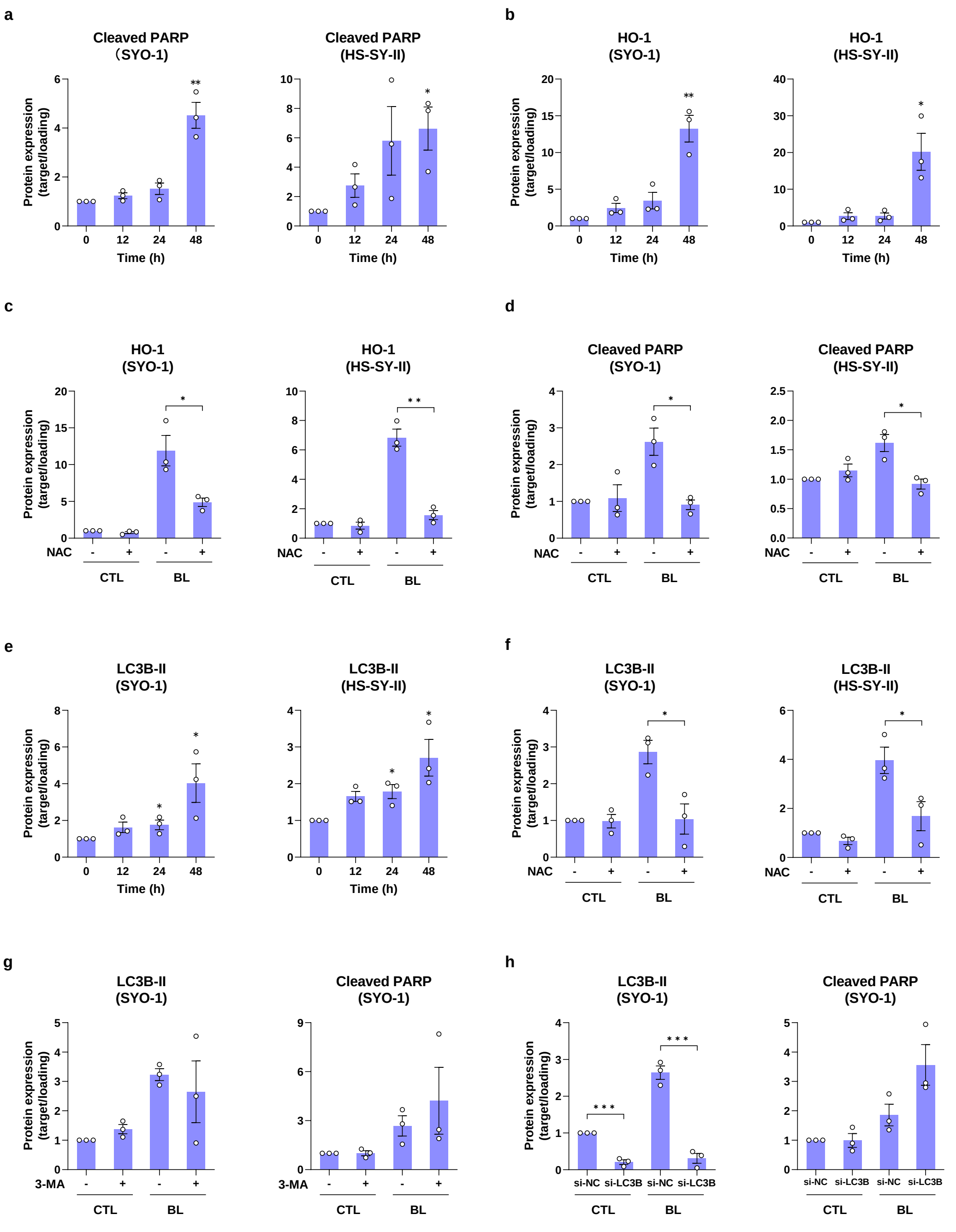

Supplementary Figure 7. Quantification of western blot bands in the main figures. (a, b, e) SYO-1 and HS-SY-II cells were irradiated with BL (0.6 mW/cm^2) for the indicated times. (c, d, f) SYO-1 and HS-SY-II cells were irradiated with BL (0.6 mW/cm^2) with or without N-acetyl-cysteine (NAC, 5 mM). (g) SYO-1 cells were irradiated with BL (0.6 mW/cm^2) with or without 3-meghylademine (3-MA, 5 mM). (h) SYO-1 cells were irradiated with BL (0.6 mW/cm^2) after si-NC or si-LC3B treatment. Data are presented as the mean ± SEM of three independent experiments. *P < 0.05, **P < 0.01, ***P < 0.001.
