# Supplementary material for: Blue light induces apoptosis and autophagy by promoting ROS‐mediated mitochondrial dysfunction in synovial sarcoma
Source: Cancer Med. 2023 Feb 1;12(8):9668–83. doi: 10.1002/cam4.5664 (PMC10166932; doi:10.1002/cam4.5664)
Supplement: Supplementary file 1 — Data S1 [file CAM4-12-9668-s001.zip › cam45664-sup-0002-SupplementaryS1.docx]

**Supplementary Materials and Methods**

***Cell viability assay***

Cell viability was determined using the Cell Counting Kit-8 (CCK-8; Dojindo) according to the manufacturer’s protocol. The cells were seeded in 96-well plates at a density of 1 × 10^4^ cells per well, incubated overnight until attachment, and exposed to light with various light intensities for the indicated time periods. Then the medium was replaced with 100 μL medium containing 10 μL CCK-8, and the cells were incubated for 1–4 h. The absorbance was measured at 450 nm with a microplate reader (Varioskan Flash; Thermo Fisher Scientific).

***Colony formation assay***

Cells were seeded in 6-well plates at a density of 1 × 10^3^ cells per well. The cells were irradiated with BL (0.1 mW/cm^2^) for 24 h. Then the cells were cultured continuously for 14 days. Finally, the cells were fixed in 100% methanol, stained with 0.5% crystal violet (Sigma-Aldrich) in 20% methanol, and photographed.

***Wound healing assay***

Cells were seeded on a culture-insert (ibidi culture-insert 2 well; ibidi GmbH) at a density of 5 × 10^5^ cells/mL. After allowing the cells to attach overnight, the culture-insert was removed, followed by BL irradiation (0.6 mW/cm^2^). Wound areas were photographed at the indicated times, and the wound healing rate was calculated under a light microscope (Nikon). Healing rate = (width of the wound at × h – width of the wound at 0 h)/width of the wound at 0 h.

***Transwell migration and invasion assay***

Filter inserts that fit into 24-well chambers (Corning) were used for the migration assay. The cells were seeded in the upper chamber at a density of 1 × 10^5^ cells/0.5 mL in serum-free medium. Then, 0.75 mL 10% FBS-supplemented medium was added to the lower chamber. After irradiation with BL, the non-migrating cells were gently removed from the surface of the chamber with cotton swabs. Next, the migrating cells on the chamber bottom were fixed and stained using Diff-Quik solutions (Sysmex). After the filter was dried, the invaded cells were imaged and counted under a light microscope. In the invasion assay, the cells were seeded in the Matrigel-coated inserts (Corning) at a density of 1 × 10^5^ cells/0.5 mL and allowed to invade under BL irradiation. The invasive cells were subsequently stained and analyzed as described above.

***Microarray analysis***

The total RNA of SYO-1 cells incubated with or without BL irradiation (0.6 mW/cm^2^) for 48 h was extracted using the RNeasy kit (Qiagen) from quadruplicate samples. The RNA samples were labeled with cyanine-3 (cRNA) using the Low Input Quick Amp Labeling Kit (Agilent Technologies). The cRNAs were applied to the slides and analyzed on the SurePrint G3 Human GE Microarray 8×60 K (Agilent Technologies). Quantile normalization and the creation of a cluster heat map were performed using GeneSpring GX software (Agilent Technologies). The volcano plot and bubble plot were made by GraphPad Prism 9 (GraphPad Software). The sample data file was imported into gene set enrichment analysis (GSEA) software (<https://www.gsea-msigdb.org/gsea/index.jsp>).

***Apoptosis assay***

Dead Cell Apoptosis Kits with Annexin V for Flow Cytometry (Thermo Fisher Scientific) were used to measure cell apoptosis. Briefly, cells were plated in a 6-well plate at a density of 1 × 10^5^ cells per well and irradiated with BL (0.6 mW/cm^2^) for the indicated times. Then the cells were harvested and washed twice with PBS. The cells were incubated with Annexin V-FITC and propidium iodide (PI) for 15 min in the dark at room temperature, and stained apoptotic cells were counted with flow cytometry (FC; BD FACSVerse; Becton Dickinson).

***Caspase activity assay***

Caspase activation was studied using the CellEvent Caspase-3/7 detection reagent (Thermo Fisher Scientific). Cells were seeded in a 6-well plate at a density of 1 × 10^5^ cells per well, incubated overnight, and irradiated with BL (0.6 mW/cm^2^) for the indicated times. The cells were harvested, washed twice with PBS, and then incubated with CellEvent reagent for 30 min in the dark at 37°C. Finally, the cells were harvested and analyzed by FC.

***Western blot analysis***

Cells were harvested with a scraper and lysed in RIPA buffer supplemented with protease and phosphatase inhibitors (Thermo Fisher Scientific). After incubation on ice for 30 min, the lysates were centrifuged at 15,000 rpm for 15 min at 4°C, and then the supernatant was moved to a new tube for analysis of the protein concentrations by the BCA Protein Assay Kit (Takara, Shiga, Japan). Collected proteins were subjected to 8%, 10%, or 15% sodium dodecyl sulfate-polyacrylamide gel electrophoresis and transferred to 0.45 μm PVDF membranes (Millipore, MA). After blocking, the membranes were incubated with the following primary antibodies: poly (ADP-ribose) polymerase (PARP) (#9542; Cell Signaling Technology [CST), heme oxygenase-1 (HO-1) (E3F4S, #43966; CST), light chain 3B (LC3B) (#2775; CST), caspase-3 (#9662; CST), and α/β tubulin (#2148; CST), all of them diluted 1:1000. The membranes were washed with Tris-buffered saline with 0.1% Tween buffer and incubated with diluted anti-rabbit IgG horseradish peroxidase-linked secondary antibody (1:3000, #7074; CST). Blots were visualized using Amersham ECL Prime (Cytiva, Tokyo, Japan). The FUSION FX. EDGE System (Vilber Lourmat) was used for the imaging, and protein levels were based on the signal intensity.

***Measurement of reactive oxygen species***

The CellROX reagent (Thermo Fisher Scientific) for detecting intracellular (total) reactive oxygen species (ROS) and MitoSOX Red reagent (Thermo Fisher Scientific) for detecting mitochondrial ROS were used according to the manufacturer’s instructions. Briefly, cells were seeded in a 6-well plate at 1.0 × 10^5^ cells per well and incubated overnight with or without BL irradiation (0.6 mW/cm^2^) for 48 h. The cells were treated with CellROX (1 μM) for 1 h or MitoSOX (5 μM) for 30 min and then harvested for FC.

***Quantitative PCR analysis***

Total RNA was extracted using the RNeasy kit (Qiagen). RNA from each sample was used to synthesize complementary DNA using the iScript cDNA Synthesis Kit (Bio-Rad) according to the manufacturer’s protocol. For quantitative PCR (qPCR), we used the Power SYBR Green Master Mix (Thermo Fisher Scientific) and the results were normalized to 18S ribosomal RNA. The primers used are listed in Table S4.

***Determination of the oxygen consumption rate***

The oxygen consumption rate (OCR) was measured using the Seahorse XF Cell Mito Stress assay on the Seahorse XF HS Mini Analyzer (Agilent Technologies) according to the manufacturer’s instructions. Briefly, cells were seeded and incubated overnight with or without BL irradiation. Before measurements, plates were equilibrated in a CO_2_-free incubator at 37°C for 1 h. Analysis was performed using 1.5 μM oligomycin, 2.0 μM carbonyl cyanide-4-(trifluoromethoxy) phenylhydrazone, and 0.5 μM rotenone/antimycin A as indicated. Data were analyzed using the Seahorse XF Cell Mito Stress Test Report generator software (Agilent Technologies). Data were normalized to the actual cell count using Hoechst 33342 (Thermo Fisher Scientific) staining immediately after OCR recording.

***Measurement of mitochondrial membrane potential***

Mitochondrial membrane potential was measured using the JC-1 MitoMP Detection Kit (Dojindo) according to the manufacturer’s protocol. In brief, cells were seeded in a 6-well plate at a density of 1.0 × 10^5^ cells per well, incubated overnight, and irradiated with BL (0.6 mW/cm^2^) for the indicated times. Then, the cells were harvested, washed twice with PBS, and stained with JC-1 at 37°C for 30 min in the dark. Subsequently, stained cells were washed, resuspended, and subjected to FC.

***Autophagy assay***

The CYTO-ID Autophagy Detection Kit (Enzo Life Sciences) was used. Cells were seeded in a 6-well plate at a density of 1.0 × 10^5^ cells per well, incubated overnight, and irradiated with BL (0.6 mW/cm^2^) for the indicated times. Then, the cells were collected and incubated with CYTO-ID detection regent for autophagic vesicle staining at 37°C for 30 min in the dark. Subsequently, stained cells were washed, resuspended, and subjected to FC.

***Small interfering RNA transfection***

The Silencer Select Predesigned small interfering RNA (siRNA) (ID: s224886 for human LC3B) was purchased from Ambion. Negative control siRNA was purchased from Nippon Gene. The si-LC3B (final concentration, 10 nM) was combined with Lipofectamine RNAiMAX Transfection Reagent (Thermo Fisher Scientific) and mixed for 15 min in each well, followed by the addition of suspension cells. The cells were incubated overnight and the medium was replaced with fresh one. The inhibition efficiency was detected by western blotting to confirm.

***Histology and immunohistochemistry***

Removed tissues were fixed in 4% paraformaldehyde for 48 h and embedded in paraffin blocks. Three-micron-thick sections were mounted on glass slides, deparaffinized, rehydrated, and stained with H&E using standard protocols. Immunohistochemistry was performed with an automated staining system using DAKO Autostainer Link48 (Agilent Technologies). Following pretreatment with the EnVision FLEX Target Retrieval Solution, High pH (Agilent Technologies), unstained 4 µm-thick sections were incubated with a rabbit monoclonal antibody against cleaved caspase-3 (Asp175) (1:500, clone 5A1E, #9664; CST) and a mouse monoclonal antibody against Ki-67 (1:100, clone MIB-1; Dako). Signals were detected using the Envision FLEX DAB+ (Agilent Technologies), and the sections were counterstained with Envision FLEX hematoxylin (Agilent Technologies).

***TUNEL analysis***

Apoptosis detection was identified using the ApopTag Plus Peroxidase In Situ Apoptosis Kit (Millipore) according to the manufacturer’s instructions. Briefly, sections were initially incubated with the TdT enzyme, which links digoxigenin-dNTP to apoptotic DNA fragments. Anti-digoxigenin antibody conjugated with peroxidase was applied to detect the digoxigenin-dNTP tails.
