# Supplementary material for: Blue light induces apoptosis and autophagy by promoting ROS‐mediated mitochondrial dysfunction in synovial sarcoma
Source: Cancer Med. 2023 Feb 1;12(8):9668–83. doi: 10.1002/cam4.5664 (PMC10166932; doi:10.1002/cam4.5664)
Supplement: Supplementary file 1 — Data S1 [file CAM4-12-9668-s001.zip › cam45664-sup-0003-Tables.docx]

**Supplementary Table 1.**

Clinicopathological data of the two patients with synovial sarcoma from whom the samples in this study were derived.

| **Patient** | **Age (y)** | **Sex** | **Location** | **Size (cm)** | **Distant metastasis** | **Stage** | **Neo adjuvant chemotherapy** | **Histology: subtype** | **IHC: SS18-SSX** |
| --- | --- | --- | --- | --- | --- | --- | --- | --- | --- |
| P1 | 42 | M | Chest wall | 11.2 | No | IIIB | AI (DXR + IFO) | Biphasic | Positive |
| P2 | 59 | M | Ankle | 7.5 | No | IIIA | No | Poorly differentiated | Positive |

y: year, M: male, DXR: doxorubicin, IFO: ifosfamide, IHC: immunohistochemistry

**Supplementary Table 2.** Reagents used in this study.

| **Reagents** | **Source** | **Catalog Number** |
| --- | --- | --- |
| Seahorse XF Cell Mito Stress Test Kit | Agilent Technologies (Santa Clara, CA) | 103010-100 |
| DMEM assay midium pack | Agilent Technologies (Santa Clara, CA) | 103680-100 |
| EnVision FLEX Target Retrieval Solution, High PH | Agilent Technologies (Santa Clara, CA) | K8004 |
| EnVision FLEX DAB+ | Agilent Technologies (Santa Clara, CA) | GV825 |
| Envision FLEX hematoxylin | Agilent Technologies (Santa Clara, CA) | K8008 |
| SurePrint G3 Human GE Microarray 8x60K Ver. 3.0 | Agilent Technologies (Santa Clara, CA) | G4858A#72363 |
| RNA Spike In Kit | Agilent Technologies (Santa Clara, CA) | 5188-5282 |
| Low Input Quick-Amp Labeling Kit | Agilent Technologies (Santa Clara, CA) | 5190-2305 |
| Gene Expression Hybridization Kit | Agilent Technologies (Santa Clara, CA) | 5188-5242 |
| GE Wash Pack | Agilent Technologies (Santa Clara, CA) | 5188-5327 |
| Silencer Select Pre-designed siRNA for human LC3B | Ambion (Grand Island, NY) | s224886 |
| 4x Laemmli Sample Buffer | Bio-Rad (Hercules, CA) | 1610747 |
| 2-Mercaptoethanol | Bio-Rad (Hercules, CA) | 1610710 |
| 10x Tris/Glycine/SDS | Bio-Rad (Hercules, CA) | 1610732 |
| 10x Tris/Glycine | Bio-Rad (Hercules, CA) | 1610734 |
| 10x TBS | Bio-Rad (Hercules, CA) | 1706435 |
| 10% Tween 20 | Bio-Rad (Hercules, CA) | 1610781 |
| 30% Acrylamide/Bis Solution | Bio-Rad (Hercules, CA) | 1610158 |
| Resolving Gel Buffer for PAGE | Bio-Rad (Hercules, CA) | 1610798 |
| Stacking Gel Buffer for PAGE | Bio-Rad (Hercules, CA) | 1610799 |
| Ammonium Persulfate (APS) | Bio-Rad (Hercules, CA) | 1610700 |
| TEMED | Bio-Rad (Hercules, CA) | 1610800 |
| Precision Plus Protein Dual Color Standards | Bio-Rad (Hercules, CA) | 1610374 |
| Precision Plus Protein All Blue Prestained | Bio-Rad (Hercules, CA) | 1610373 |
| iScript Advanced cDNA Synthesis kit | Bio-Rad (Hercules, CA) | 1725038 |
| EvaGreen 20X in Water | Biotium (Fremont, CA) | 31000 |
| PARP Antibody | Cell Signaling Technology (Danvers, MA) | 9542 |
| HO-1 (E3F4S) | Cell Signaling Technology (Danvers, MA) | 43966 |
| LC3B Antibody | Cell Signaling Technology (Danvers, MA) | 2775 |
| Caspase-3 Antibody | Cell Signaling Technology (Danvers, MA) | 9662 |
| α/β-Tubulin Antibody | Cell Signaling Technology (Danvers, MA) | 2148 |
| Cleaved Caspase-3 (Asp175) | Cell Signaling Technology (Danvers, MA) | 9664 |
| Anti-rabbit IgG, HRP-linked Antibody | Cell Signaling Technology (Danvers, MA) | 7074S |
| ECL Prime Western Blotting Detection Reagent | Cytiva (Tokyo, Japan) | RPN2232 |
| Ki-67 Antigen | DAKO (Glostrup, Denmark) | M7240 |
| Cell Counting Kit-8 | Dojindo (Kumamoto, Japan) | 341-07624 |
| JC-1 MitoMP Detection Kit | Dojindo (Kumamoto, Japan) | MT09 |
| CYTO-ID autophagy detection kit | Enzo Life Sciences (Farmingdale, NY) | ENZ-KIT175-0200 |
| ApopTag Plus Peroxidase In Situ Apoptosis Kit | Millipore (Burlington, MA) | S7101 |
| 4%-Paraformaldehyde Phosphate Buffer Solution | Nakalai (Kyoto, Japan) | 09154-56 |
| Negative control siRNA | Nippon Gene (Tokyo, Japan) | 211124 |
| RNeasy Mini Kit (50) | QIAGEN (Hilden, Germany) | 74104 |
| Phosphate Buffered Saline | Santa Cruz Biotechnology (Santa Cruz, CA) | |
| 3-Methyladenine | Selleckchem (Houston, TX) | 5142-23-4 |
| Dulbecco’s Modified Eagle’s Medium | Sigma-Aldrich (St.Louis, MO) | D6046 |
| Fetal Bovine Serum | Sigma-Aldrich (St.Louis, MO) | F7524 |
| Penicillin-Streptomycin | Sigma-Aldrich (St.Louis, MO) | D6046 |
| Collagenase from Clostridium h | Sigma-Aldrich (St.Louis, MO) | C2139 |
| Crystal violet solution | Sigma-Aldrich (St.Louis, MO) | V5265 |
| N-Acetyl-L-cysteine | Sigma-Aldrich (St.Louis, MO) | A7250 |
| Protease Inhibitor Cocktail | Sigma-Aldrich (St.Louis, MO) | I3786 |
| Phosphatase Inhibitor Cocktail | Sigma-Aldrich (St.Louis, MO) | P0044 |
| Reference Dye for Quantitative PCR 100 ×, solution | Sigma-Aldrich (St.Louis, MO) | R4526 |
| CELLBANKER 1 | Takara (Shiga, Japan) | CB011 |
| BCA Protein Assay Kit | Takara (Shiga, Japan) | T9300A |
| Trypsin-EDTA (0.05%) | Thermo Fisher Scientific (Waltham, MA) | 25300062 |
| Dead Cell Apoptosis Kit with Annexin V FITC and PI | Thermo Fisher Scientific (Waltham, MA) | V13242 |
| CellEven Caspase-3/7 Green Flow Cytometry Assay Kit | Thermo Fisher Scientific (Waltham, MA) | C10427 |
| FxCycle PI/RNase Staining Solution | Thermo Fisher Scientific (Waltham, MA) | F10797 |
| RIPA Lysis and Extraction Buffer | Thermo Fisher Scientific (Waltham, MA) | 89900 |
| CellROX Orange Flow Cytometry Assay Kit | Thermo Fisher Scientific (Waltham, MA | C10493 |
| MitoSOX Red Mitochondrial Superoxide Indicator | Thermo Fisher Scientific (Waltham, MA) | M36008 |
| Hanks′ Balanced Salt solution | Thermo Fisher Scientific (Waltham, MA) | 14025092 |
| Power SYBR Green PCR Master Mix | Thermo Fisher Scientific (Waltham, MA) | 4368706 |
| Hoechst 33342 | Thermo Fisher Scientific (Waltham, MA) | H3570 |
| Lipofectamine RNAiMAX Transfection Reagent | Thermo Fisher Scientific (Waltham, MA) | 13778030 |
| CTS Opti-MEM I Medium | Thermo Fisher Scientific (Waltham, MA) | A4124801 |
| Intracellular ATP assay kit ver.2 | Toyo B-Net (Tokyo, Japan) | 382-14581 |
| Methanol | Wako (Osaka, Japan) | 137-01823 |
| Sodium Dodecyl Sulfate | Wako (Osaka, Japan) | 194-13985 |

**Supplementary Table 3.** The top upregulated or downregulated gene sets based on NES >1.5 or <−1.5, and a normal *p*-value of <0.05 as the threshold. NES: normalized enrichment score; NOM, nominal; FDR: false discovery rate.

**Hallmark**

| **NAME** | **NES** | **NOM p-val** | **FDR q-val** |
| --- | --- | --- | --- |
| HALLMARK_G2M_CHECKPOINT | -3.12 | 0.000 | 0.000 |
| HALLMARK_E2F_TARGETS | -3.05 | 0.000 | 0.000 |
| HALLMARK_MYC_TARGETS_V2 | -2.87 | 0.000 | 0.000 |
| HALLMARK_P53_PATHWAY | 2.67 | 0.000 | 0.000 |
| HALLMARK_MYC_TARGETS_V1 | -2.62 | 0.000 | 0.000 |
| HALLMARK_TNFA_SIGNALING_VIA_NFKB | 2.40 | 0.000 | 0.000 |
| HALLMARK_MITOTIC_SPINDLE | -2.36 | 0.000 | 0.000 |
| HALLMARK_REACTIVE_OXYGEN_SPECIES_PATHWAY | 2.23 | 0.000 | 0.000 |
| HALLMARK_APOPTOSIS | 2.16 | 0.000 | 0.000 |
| HALLMARK_CHOLESTEROL_HOMEOSTASIS | 2.15 | 0.000 | 0.000 |
| HALLMARK_HEME_METABOLISM | 2.02 | 0.000 | 0.000 |
| HALLMARK_HYPOXIA | 1.95 | 0.000 | 0.000 |
| HALLMARK_XENOBIOTIC_METABOLISM | 1.87 | 0.000 | 0.001 |
| HALLMARK_INTERFERON_ALPHA_RESPONSE | -1.76 | 0.001 | 0.013 |
| HALLMARK_UNFOLDED_PROTEIN_RESPONSE | -1.60 | 0.003 | 0.092 |
| HALLMARK_BILE_ACID_METABOLISM | 1.59 | 0.000 | 0.015 |
| HALLMARK_SPERMATOGENESIS | -1.57 | 0.003 | 0.140 |

**Gene Ontology**

| **NAME** | **NES** | **NOM p-val** | **FDR q-val** |
| --- | --- | --- | --- |
| GOCC_DNA_PACKAGING_COMPLEX | -3.08 | 0.000 | 0.000 |
| GOBP_NUCLEOSOME_ASSEMBLY | -3.04 | 0.000 | 0.000 |
| GOBP_NUCLEOSOME_ORGANIZATION | -2.93 | 0.000 | 0.000 |
| GOBP_MITOTIC_SISTER_CHROMATID_SEGREGATION | -2.82 | 0.000 | 0.000 |
| GOBP_DNA_CONFORMATION_CHANGE | -2.81 | 0.000 | 0.000 |
| GOBP_DNA_REPLICATION_DEPENDENT_CHROMATIN_ORGANIZATION | -2.79 | 0.000 | 0.000 |
| GOBP_PROTEIN_DNA_COMPLEX_ASSEMBLY | -2.79 | 0.000 | 0.000 |
| GOCC_PROTEIN_DNA_COMPLEX | -2.79 | 0.000 | 0.000 |
| GOBP_DNA_PACKAGING | -2.79 | 0.000 | 0.000 |
| GOBP_PROTEIN_DNA_COMPLEX_SUBUNIT_ORGANIZATION | -2.77 | 0.000 | 0.000 |
| GOBP_SISTER_CHROMATID_SEGREGATION | -2.76 | 0.000 | 0.000 |
| GOCC_NUCLEAR_CHROMOSOME | -2.76 | 0.000 | 0.000 |
| GOCC_CHROMOSOME_CENTROMERIC_REGION | -2.73 | 0.000 | 0.000 |
| GOCC_PRERIBOSOME | -2.72 | 0.000 | 0.000 |
| GOBP_CHROMATIN_ASSEMBLY_OR_DISASSEMBLY | -2.71 | 0.000 | 0.000 |
| GOBP_RIBOSOME_BIOGENESIS | -2.69 | 0.000 | 0.000 |
| GOBP_REGULATION_OF_CHROMOSOME_SEPARATION | -2.66 | 0.000 | 0.000 |
| GOBP_MITOTIC_NUCLEAR_DIVISION | -2.66 | 0.000 | 0.000 |
| GOBP_NUCLEAR_CHROMOSOME_SEGREGATION | -2.63 | 0.000 | 0.000 |
| GOCC_CHROMOSOMAL_REGION | -2.62 | 0.000 | 0.000 |
| GOBP_DNA_REPLICATION_INDEPENDENT_CHROMATIN_ORGANIZATION | -2.62 | 0.000 | 0.000 |
| GOBP_REGULATION_OF_MITOTIC_SISTER_CHROMATID_SEGREGATION | -2.61 | 0.000 | 0.000 |
| GOBP_CHROMOSOME_SEGREGATION | -2.61 | 0.000 | 0.000 |
| GOBP_RRNA_METABOLIC_PROCESS | -2.59 | 0.000 | 0.000 |
| GOBP_RIBONUCLEOPROTEIN_COMPLEX_BIOGENESIS | -2.59 | 0.000 | 0.000 |
| GOBP_CHROMOSOME_SEPARATION | -2.58 | 0.000 | 0.000 |
| GOBP_NEGATIVE_REGULATION_OF_MEGAKARYOCYTE_DIFFERENTIATION | -2.58 | 0.000 | 0.000 |
| GOBP_CHROMATIN_REMODELING | -2.57 | 0.000 | 0.000 |
| GOBP_NEGATIVE_REGULATION_OF_CHROMOSOME_ORGANIZATION | -2.54 | 0.000 | 0.000 |
| GOBP_REGULATION_OF_CHROMOSOME_SEGREGATION | -2.53 | 0.000 | 0.000 |
| GOBP_METAPHASE_ANAPHASE_TRANSITION_OF_CELL_CYCLE | -2.52 | 0.000 | 0.000 |
| GOCC_CHROMOSOME_CENTROMERIC_CORE_DOMAIN | -2.50 | 0.000 | 0.000 |
| GOBP_REGULATION_OF_CHROMOSOME_ORGANIZATION | -2.50 | 0.000 | 0.000 |
| GOBP_NEGATIVE_REGULATION_OF_METAPHASE_ANAPHASE_TRANSITION_OF_CELL_CYCLE | -2.49 | 0.000 | 0.000 |
| GOCC_CONDENSED_CHROMOSOME | -2.46 | 0.000 | 0.000 |
| GOBP_NEGATIVE_REGULATION_OF_NUCLEAR_DIVISION | -2.46 | 0.000 | 0.000 |
| GOBP_CHROMOSOME_CONDENSATION | -2.44 | 0.000 | 0.000 |
| GOBP_MICROTUBULE_CYTOSKELETON_ORGANIZATION_INVOLVED_IN_MITOSIS | -2.44 | 0.000 | 0.000 |
| GOCC_SECONDARY_LYSOSOME | 2.44 | 0.000 | 0.001 |
| GOBP_NCRNA_PROCESSING | -2.43 | 0.000 | 0.000 |
| GOBP_RIBOSOMAL_SMALL_SUBUNIT_BIOGENESIS | -2.43 | 0.000 | 0.000 |
| GOCC_CONDENSED_CHROMOSOME_CENTROMERIC_REGION | -2.42 | 0.000 | 0.000 |
| GOBP_CHROMOSOME_LOCALIZATION | -2.38 | 0.000 | 0.000 |
| GOBP_METAPHASE_PLATE_CONGRESSION | -2.38 | 0.000 | 0.000 |
| GOBP_REGULATION_OF_MITOTIC_NUCLEAR_DIVISION | -2.37 | 0.000 | 0.000 |
| GOBP_ORGANELLE_FISSION | -2.37 | 0.000 | 0.000 |
| GOBP_DNA_DEPENDENT_DNA_REPLICATION | -2.36 | 0.000 | 0.000 |
| GOBP_DNA_GEOMETRIC_CHANGE | -2.35 | 0.000 | 0.000 |
| GOBP_TELOMERE_ORGANIZATION | -2.35 | 0.000 | 0.000 |
| GOMF_SNORNA_BINDING | -2.35 | 0.000 | 0.000 |
| GOBP_SPINDLE_ORGANIZATION | -2.35 | 0.000 | 0.000 |
| GOCC_SPINDLE_POLE | -2.35 | 0.000 | 0.000 |
| GOCC_90S_PRERIBOSOME | -2.34 | 0.000 | 0.000 |
| GOBP_NCRNA_METABOLIC_PROCESS | -2.33 | 0.000 | 0.000 |
| GOCC_PRERIBOSOME_LARGE_SUBUNIT_PRECURSOR | -2.33 | 0.000 | 0.000 |
| GOBP_RIBOSOMAL_LARGE_SUBUNIT_BIOGENESIS | -2.33 | 0.000 | 0.000 |
| GOMF_NUCLEOSOMAL_DNA_BINDING | -2.32 | 0.000 | 0.000 |
| GOCC_AUTOPHAGOSOME | 2.32 | 0.000 | 0.004 |
| GOBP_RIBOSOME_ASSEMBLY | -2.32 | 0.000 | 0.000 |
| GOMF_SINGLE_STRANDED_DNA_HELICASE_ACTIVITY | -2.31 | 0.000 | 0.000 |
| GOBP_DNA_STRAND_ELONGATION | -2.30 | 0.000 | 0.000 |
| GOBP_REGULATION_OF_NUCLEAR_DIVISION | -2.29 | 0.000 | 0.000 |
| GOBP_MATURATION_OF_SSU_RRNA | -2.29 | 0.000 | 0.000 |
| GOCC_CHROMOSOME_TELOMERIC_REGION | -2.28 | 0.000 | 0.000 |
| GOBP_MITOTIC_SPINDLE_ORGANIZATION | -2.28 | 0.000 | 0.000 |
| GOBP_CELLULAR_OXIDANT_DETOXIFICATION | 2.28 | 0.000 | 0.006 |
| GOMF_HELICASE_ACTIVITY | -2.27 | 0.000 | 0.000 |
| GOBP_DNA_REPLICATION | -2.27 | 0.000 | 0.000 |
| GOBP_CENTROMERE_COMPLEX_ASSEMBLY | -2.27 | 0.000 | 0.000 |
| GOBP_MATURATION_OF_5_8S_RRNA | -2.27 | 0.000 | 0.000 |
| GOMF_CATALYTIC_ACTIVITY_ACTING_ON_RNA | -2.26 | 0.000 | 0.000 |
| GOBP_MATURATION_OF_SSU_RRNA_FROM_TRICISTRONIC_RRNA_TRANSCRIPT_SSU_RRNA_5_8S_RRNA_LSU_RRNA | -2.26 | 0.000 | 0.000 |
| GOMF_PROTEIN_HETERODIMERIZATION_ACTIVITY | -2.26 | 0.000 | 0.000 |
| GOBP_RNA_SPLICING_VIA_TRANSESTERIFICATION_REACTIONS | -2.25 | 0.000 | 0.000 |
| GOCC_SPLICEOSOMAL_COMPLEX | -2.25 | 0.000 | 0.000 |
| GOBP_PROTEIN_LOCALIZATION_TO_CHROMOSOME | -2.25 | 0.000 | 0.000 |
| GOCC_SMALL_SUBUNIT_PROCESSOME | -2.25 | 0.000 | 0.000 |
| GOCC_SPINDLE | -2.24 | 0.000 | 0.001 |
| GOMF_ATP_DEPENDENT_ACTIVITY_ACTING_ON_DNA | -2.23 | 0.000 | 0.001 |
| GOBP_ATTACHMENT_OF_SPINDLE_MICROTUBULES_TO_KINETOCHORE | -2.23 | 0.000 | 0.001 |
| GOMF_DNA_HELICASE_ACTIVITY | -2.23 | 0.000 | 0.001 |
| GOBP_REGULATION_OF_DNA_DEPENDENT_DNA_REPLICATION | -2.23 | 0.000 | 0.001 |
| GOBP_SISTER_CHROMATID_COHESION | -2.23 | 0.000 | 0.001 |
| GOBP_POSITIVE_REGULATION_OF_CELL_CYCLE_PROCESS | -2.23 | 0.000 | 0.002 |
| GOBP_DNA_UNWINDING_INVOLVED_IN_DNA_REPLICATION | -2.22 | 0.000 | 0.002 |
| GOBP_MITOTIC_METAPHASE_PLATE_CONGRESSION | -2.22 | 0.000 | 0.003 |
| GOBP_NEGATIVE_REGULATION_OF_RNA_SPLICING | -2.22 | 0.000 | 0.003 |
| GOMF_FERROUS_IRON_BINDING | 2.22 | 0.000 | 0.011 |
| GOMF_ANTIOXIDANT_ACTIVITY | 2.21 | 0.000 | 0.009 |
| GOMF_ATP_DEPENDENT_ACTIVITY_ACTING_ON_RNA | -2.21 | 0.000 | 0.003 |
| GOBP_RIBOSOMAL_LARGE_SUBUNIT_ASSEMBLY | -2.21 | 0.000 | 0.003 |
| GOBP_RNA_SPLICING | -2.21 | 0.000 | 0.003 |
| GOBP_DNA_STRAND_ELONGATION_INVOLVED_IN_DNA_REPLICATION | -2.21 | 0.000 | 0.003 |
| GOBP_PROTEIN_LOCALIZATION_TO_CHROMOSOME_CENTROMERIC_REGION | -2.20 | 0.000 | 0.004 |
| GOBP_CLEAVAGE_INVOLVED_IN_RRNA_PROCESSING | -2.20 | 0.000 | 0.004 |
| GOBP_REGULATION_OF_MEGAKARYOCYTE_DIFFERENTIATION | -2.20 | 0.000 | 0.004 |
| GOBP_ESTABLISHMENT_OF_MITOTIC_SPINDLE_LOCALIZATION | -2.20 | 0.000 | 0.005 |
| GOBP_MATURATION_OF_5_8S_RRNA_FROM_TRICISTRONIC_RRNA_TRANSCRIPT_SSU_RRNA_5_8S_RRNA_LSU_RRNA | -2.20 | 0.000 | 0.005 |
| GOBP_PROSTANOID_BIOSYNTHETIC_PROCESS | 2.19 | 0.000 | 0.012 |
| GOBP_RESPONSE_TO_TYPE_I_INTERFERON | -2.19 | 0.000 | 0.006 |
| GOMF_NUCLEOSOME_BINDING | -2.19 | 0.000 | 0.006 |
| GOBP_PROTEIN_LOCALIZATION_TO_CONDENSED_CHROMOSOME | -2.18 | 0.000 | 0.006 |
| GOBP_MEIOTIC_SPINDLE_ORGANIZATION | -2.18 | 0.000 | 0.006 |
| GOCC_PRECATALYTIC_SPLICEOSOME | -2.18 | 0.000 | 0.006 |
| GOCC_EXORIBONUCLEASE_COMPLEX | -2.17 | 0.000 | 0.008 |
| GOCC_SPINDLE_MIDZONE | -2.17 | 0.000 | 0.009 |
| GOBP_TRNA_METABOLIC_PROCESS | -2.16 | 0.000 | 0.011 |
| GOCC_MITOTIC_SPINDLE | -2.15 | 0.000 | 0.011 |
| GOBP_RNA_LOCALIZATION | -2.15 | 0.000 | 0.012 |
| GOCC_SPLICEOSOMAL_TRI_SNRNP_COMPLEX | -2.15 | 0.000 | 0.012 |
| GOBP_DNA_REPLICATION_INITIATION | -2.15 | 0.000 | 0.012 |
| GOBP_RIBONUCLEOPROTEIN_COMPLEX_SUBUNIT_ORGANIZATION | -2.15 | 0.000 | 0.013 |
| GOBP_NEGATIVE_REGULATION_OF_MRNA_SPLICING_VIA_SPLICEOSOME | -2.14 | 0.000 | 0.016 |
| GOBP_MRNA_PROCESSING | -2.14 | 0.000 | 0.018 |
| GOBP_SPINDLE_LOCALIZATION | -2.14 | 0.000 | 0.019 |
| GOBP_NUCLEOSOME_POSITIONING | -2.14 | 0.000 | 0.020 |
| GOCC_U2_TYPE_SPLICEOSOMAL_COMPLEX | -2.14 | 0.000 | 0.021 |
| GOMF_ATP_HYDROLYSIS_ACTIVITY | -2.13 | 0.000 | 0.021 |
| GOBP_RRNA_MODIFICATION | -2.13 | 0.000 | 0.021 |
| GOBP_PROSTANOID_METABOLIC_PROCESS | 2.13 | 0.000 | 0.025 |
| GOBP_FEMALE_MEIOTIC_NUCLEAR_DIVISION | -2.12 | 0.000 | 0.024 |
| GOBP_MITOTIC_CELL_CYCLE_PHASE_TRANSITION | -2.12 | 0.000 | 0.024 |
| GOBP_BONE_RESORPTION | 2.12 | 0.000 | 0.024 |
| GOCC_PRERIBOSOME_SMALL_SUBUNIT_PRECURSOR | -2.12 | 0.000 | 0.024 |
| GOBP_MACROAUTOPHAGY | 2.12 | 0.000 | 0.022 |
| GOBP_MEIOTIC_CHROMOSOME_SEGREGATION | -2.11 | 0.000 | 0.025 |
| GOBP_MEGAKARYOCYTE_DIFFERENTIATION | -2.11 | 0.000 | 0.025 |
| GOBP_SPINDLE_ASSEMBLY | -2.10 | 0.000 | 0.030 |
| GOBP_KINETOCHORE_ORGANIZATION | -2.10 | 0.000 | 0.032 |
| GOBP_REGULATION_OF_CELL_CYCLE_PHASE_TRANSITION | -2.10 | 0.000 | 0.036 |
| GOBP_MRNA_EXPORT_FROM_NUCLEUS | -2.09 | 0.000 | 0.036 |
| GOMF_CATALYTIC_ACTIVITY_ACTING_ON_A_TRNA | -2.09 | 0.000 | 0.037 |
| GOBP_CELL_CYCLE_CHECKPOINT_SIGNALING | -2.09 | 0.000 | 0.038 |
| GOBP_CELL_CYCLE_PHASE_TRANSITION | -2.09 | 0.000 | 0.038 |
| GOBP_RESPONSE_TO_INTERFERON_BETA | -2.09 | 0.000 | 0.039 |
| GOBP_MEIOTIC_CELL_CYCLE_PROCESS | -2.09 | 0.000 | 0.042 |
| GOBP_REGULATION_OF_MITOTIC_CELL_CYCLE_PHASE_TRANSITION | -2.09 | 0.000 | 0.042 |
| GOBP_AUTOPHAGOSOME_ORGANIZATION | 2.08 | 0.000 | 0.033 |
| GOCC_HETEROCHROMATIN | -2.08 | 0.000 | 0.049 |
| GOBP_ESTABLISHMENT_OF_SPINDLE_ORIENTATION | -2.08 | 0.000 | 0.049 |
| GOBP_GLUTATHIONE_METABOLIC_PROCESS | 2.07 | 0.000 | 0.035 |
| GOBP_DNA_REPAIR | -2.07 | 0.000 | 0.052 |
| GOMF_CATALYTIC_ACTIVITY_ACTING_ON_DNA | -2.07 | 0.000 | 0.055 |
| GOBP_CELLULAR_RESPONSE_TO_TOXIC_SUBSTANCE | 2.06 | 0.000 | 0.035 |
| GOBP_NON_MEMBRANE_BOUNDED_ORGANELLE_ASSEMBLY | -2.06 | 0.000 | 0.064 |
| GOBP_DOUBLE_STRAND_BREAK_REPAIR | -2.06 | 0.000 | 0.066 |
| GOBP_REGULATION_OF_DNA_REPLICATION | -2.06 | 0.000 | 0.066 |
| GOCC_REPLICATION_FORK | -2.05 | 0.000 | 0.069 |
| GOBP_MRNA_TRANSPORT | -2.05 | 0.000 | 0.072 |
| GOCC_PHAGOPHORE_ASSEMBLY_SITE | 2.05 | 0.000 | 0.042 |
| GOBP_CELL_CYCLE_DNA_REPLICATION | -2.05 | 0.000 | 0.076 |
| GOBP_REGULATION_OF_DNA_TEMPLATED_TRANSCRIPTION_IN_RESPONSE_TO_STRESS | 2.04 | 0.000 | 0.042 |
| GOBP_MITOTIC_SPINDLE_ASSEMBLY | -2.04 | 0.000 | 0.092 |
| GOBP_RNA_PHOSPHODIESTER_BOND_HYDROLYSIS | -2.04 | 0.000 | 0.094 |
| GOBP_MITOTIC_CHROMOSOME_CONDENSATION | -2.04 | 0.000 | 0.094 |
| GOBP_DNA_RECOMBINATION | -2.03 | 0.000 | 0.118 |
| GOBP_MEIOTIC_CELL_CYCLE | -2.03 | 0.000 | 0.120 |
| GOBP_NEGATIVE_REGULATION_OF_MACROAUTOPHAGY | 2.03 | 0.000 | 0.047 |
| GOBP_NEGATIVE_REGULATION_OF_TYPE_I_INTERFERON_MEDIATED_SIGNALING_PATHWAY | -2.03 | 0.000 | 0.128 |
| GOBP_RESPONSE_TO_AMINO_ACID_STARVATION | 2.02 | 0.000 | 0.047 |
| GOBP_RECOMBINATIONAL_REPAIR | -2.02 | 0.000 | 0.136 |
| GOBP_NEGATIVE_REGULATION_OF_VIRAL_GENOME_REPLICATION | -2.02 | 0.000 | 0.139 |
| GOBP_POSITIVE_REGULATION_OF_CELL_CYCLE_PHASE_TRANSITION | -2.02 | 0.000 | 0.150 |
| GOBP_NEGATIVE_REGULATION_OF_CELL_CYCLE_PROCESS | -2.02 | 0.000 | 0.153 |
| GOCC_SNO_S_RNA_CONTAINING_RIBONUCLEOPROTEIN_COMPLEX | -2.02 | 0.000 | 0.153 |
| GOBP_REGULATION_OF_MITOTIC_CELL_CYCLE | -2.02 | 0.000 | 0.153 |
| GOBP_POSITIVE_REGULATION_OF_CYTOKINESIS | -2.02 | 0.000 | 0.158 |
| GOBP_ESTABLISHMENT_OF_RNA_LOCALIZATION | -2.01 | 0.000 | 0.167 |
| GOBP_POSITIVE_REGULATION_OF_CELL_CYCLE | -2.01 | 0.000 | 0.173 |
| GOBP_RNA_EXPORT_FROM_NUCLEUS | -2.01 | 0.000 | 0.183 |
| GOBP_TRNA_PROCESSING | -2.01 | 0.000 | 0.185 |
| GOBP_DETOXIFICATION | 2.00 | 0.000 | 0.059 |
| GOCC_CONDENSED_NUCLEAR_CHROMOSOME | -2.00 | 0.000 | 0.216 |
| GOMF_EXONUCLEASE_ACTIVITY_ACTIVE_WITH_EITHER_RIBO_OR_DEOXYRIBONUCLEIC_ACIDS_AND_PRODUCING_5_PHOSPHOMONOESTERS | -1.99 | 0.000 | 0.220 |
| GOCC_CATALYTIC_STEP_2_SPLICEOSOME | -1.99 | 0.000 | 0.226 |
| GOBP_NEGATIVE_REGULATION_OF_MYELOID_CELL_DIFFERENTIATION | -1.99 | 0.000 | 0.229 |
| GOCC_NUCLEOID | -1.99 | 0.000 | 0.235 |
| GOBP_CHROMOSOME_ORGANIZATION_INVOLVED_IN_MEIOTIC_CELL_CYCLE | -1.98 | 0.000 | 0.249 |
| GOBP_NUCLEOBASE_BIOSYNTHETIC_PROCESS | -1.98 | 0.000 | 0.250 |
| GOMF_DNA_REPLICATION_ORIGIN_BINDING | -1.98 | 0.000 | 0.251 |
| GOBP_NEGATIVE_REGULATION_OF_MRNA_PROCESSING | -1.98 | 0.000 | 0.254 |
| GOBP_REGULATION_OF_MRNA_SPLICING_VIA_SPLICEOSOME | -1.98 | 0.000 | 0.255 |
| GOBP_DNA_REPLICATION_CHECKPOINT_SIGNALING | -1.98 | 0.000 | 0.258 |
| GOBP_NEGATIVE_REGULATION_OF_CELL_CYCLE_PHASE_TRANSITION | -1.98 | 0.000 | 0.258 |
| GOBP_EMBRYONIC_SKELETAL_SYSTEM_MORPHOGENESIS | -1.98 | 0.000 | 0.261 |
| GOBP_REGULATION_OF_MACROAUTOPHAGY | 1.98 | 0.000 | 0.072 |
| GOBP_ESTABLISHMENT_OF_PROTEIN_LOCALIZATION_TO_CHROMOSOME | -1.98 | 0.000 | 0.268 |
| GOBP_TRANSLATIONAL_TERMINATION | -1.98 | 0.000 | 0.271 |
| GOCC_SPINDLE_MICROTUBULE | -1.98 | 0.000 | 0.273 |
| GOBP_POSITIVE_REGULATION_OF_RELEASE_OF_CYTOCHROME_C_FROM_MITOCHONDRIA | 1.97 | 0.003 | 0.072 |
| GOBP_REGULATION_OF_MICROGLIAL_CELL_ACTIVATION | 1.97 | 0.000 | 0.069 |
| GOBP_REGULATION_OF_RNA_SPLICING | -1.97 | 0.000 | 0.287 |
| GOBP_RNA_METHYLATION | -1.97 | 0.000 | 0.290 |
| GOBP_MITOTIC_CELL_CYCLE_CHECKPOINT_SIGNALING | -1.97 | 0.001 | 0.291 |
| GOBP_KINETOCHORE_ASSEMBLY | -1.97 | 0.000 | 0.302 |
| GOBP_AMYLOID_FIBRIL_FORMATION | 1.97 | 0.000 | 0.068 |
| GOBP_POSITIVE_REGULATION_OF_NEUROINFLAMMATORY_RESPONSE | 1.97 | 0.003 | 0.065 |
| GOCC_AUTOPHAGOSOME_MEMBRANE | 1.97 | 0.000 | 0.063 |
| GOBP_MATURATION_OF_LSU_RRNA | -1.97 | 0.000 | 0.315 |
| GOBP_VIRAL_TRANSCRIPTION | -1.97 | 0.001 | 0.315 |
| GOCC_CAJAL_BODY | -1.97 | 0.000 | 0.323 |
| GOBP_REGULATION_OF_UBIQUITIN_PROTEIN_LIGASE_ACTIVITY | -1.96 | 0.000 | 0.336 |
| GOBP_POSITIVE_REGULATION_OF_MACROAUTOPHAGY | 1.96 | 0.000 | 0.065 |
| GOBP_REGULATION_OF_TELOMERE_MAINTENANCE_VIA_TELOMERE_LENGTHENING | -1.96 | 0.001 | 0.363 |
| GOMF_3_5_EXONUCLEASE_ACTIVITY | -1.96 | 0.000 | 0.364 |
| GOBP_REGULATION_OF_CYTOKINESIS | -1.96 | 0.000 | 0.366 |
| GOMF_STRUCTURAL_CONSTITUENT_OF_NUCLEAR_PORE | -1.95 | 0.000 | 0.379 |
| GOBP_REGULATION_OF_TELOMERE_MAINTENANCE | -1.95 | 0.000 | 0.381 |
| GOMF_3_5_DNA_HELICASE_ACTIVITY | -1.95 | 0.000 | 0.384 |
| GOBP_REGULATION_OF_VACUOLE_ORGANIZATION | 1.95 | 0.000 | 0.069 |
| GOCC_DNA_POLYMERASE_COMPLEX | -1.95 | 0.000 | 0.386 |
| GOBP_DNA_BIOSYNTHETIC_PROCESS | -1.95 | 0.000 | 0.386 |
| GOMF_CATALYTIC_ACTIVITY_ACTING_ON_A_RRNA | -1.95 | 0.000 | 0.387 |
| GOBP_NEURON_FATE_COMMITMENT | -1.95 | 0.000 | 0.387 |
| GOMF_SINGLE_STRANDED_DNA_BINDING | -1.95 | 0.000 | 0.387 |
| GOMF_EXONUCLEASE_ACTIVITY | -1.95 | 0.000 | 0.398 |
| GOBP_POSITIVE_REGULATION_OF_CHROMOSOME_ORGANIZATION | -1.95 | 0.000 | 0.399 |
| GOBP_RRNA_TRANSCRIPTION | -1.94 | 0.002 | 0.412 |
| GOBP_REGULATION_OF_AUTOPHAGY | 1.94 | 0.000 | 0.075 |
| GOBP_RESPONSE_TO_INTERLEUKIN_4 | -1.94 | 0.002 | 0.433 |
| GOBP_NEGATIVE_REGULATION_OF_DNA_METABOLIC_PROCESS | -1.94 | 0.000 | 0.436 |
| GOBP_NUCLEIC_ACID_PHOSPHODIESTER_BOND_HYDROLYSIS | -1.94 | 0.000 | 0.442 |
| GOMF_RNA_METHYLTRANSFERASE_ACTIVITY | -1.93 | 0.000 | 0.459 |
| GOBP_DNA_DEPENDENT_DNA_REPLICATION_MAINTENANCE_OF_FIDELITY | -1.93 | 0.000 | 0.459 |
| GOBP_REGULATION_OF_BONE_RESORPTION | 1.93 | 0.000 | 0.079 |
| GOBP_RNA_MODIFICATION | -1.93 | 0.000 | 0.473 |
| GOCC_MITOTIC_SPINDLE_POLE | -1.93 | 0.000 | 0.475 |
| GOBP_REGULATION_OF_TYPE_I_INTERFERON_MEDIATED_SIGNALING_PATHWAY | -1.93 | 0.000 | 0.486 |
| GOBP_NEGATIVE_REGULATION_OF_MITOTIC_CELL_CYCLE_PHASE_TRANSITION | -1.93 | 0.000 | 0.493 |
| GOBP_REGULATION_OF_CELL_CYCLE_CHECKPOINT | -1.93 | 0.000 | 0.499 |
| GOBP_TELOMERASE_RNA_LOCALIZATION | -1.93 | 0.000 | 0.505 |
| GOBP_NUCLEOSOME_MOBILIZATION | -1.92 | 0.000 | 0.526 |
| GOCC_MIDBODY | -1.92 | 0.000 | 0.527 |
| GOBP_VACUOLAR_ACIDIFICATION | 1.92 | 0.005 | 0.091 |
| GOBP_REGULATION_OF_VIRAL_GENOME_REPLICATION | -1.91 | 0.001 | 0.580 |
| GOMF_MRNA_5_UTR_BINDING | -1.91 | 0.000 | 0.580 |
| GOBP_POSITIVE_REGULATION_OF_DNA_BIOSYNTHETIC_PROCESS | -1.91 | 0.000 | 0.580 |
| GOBP_NEUTROPHIL_HOMEOSTASIS | 1.91 | 0.000 | 0.091 |
| GOBP_REGULATION_OF_MRNA_PROCESSING | -1.91 | 0.000 | 0.587 |
| GOCC_LATE_ENDOSOME_MEMBRANE | 1.91 | 0.000 | 0.089 |
| GOBP_POSITIVE_REGULATION_OF_VACUOLE_ORGANIZATION | 1.91 | 0.003 | 0.087 |
| GOBP_RNA_PHOSPHODIESTER_BOND_HYDROLYSIS_ENDONUCLEOLYTIC | -1.91 | 0.000 | 0.605 |
| GOBP_CYTOKINESIS | -1.91 | 0.000 | 0.631 |
| GOBP_SELECTIVE_AUTOPHAGY | 1.90 | 0.000 | 0.089 |
| GOBP_NEGATIVE_REGULATION_OF_CELL_CYCLE | -1.90 | 0.000 | 0.641 |
| GOBP_NCRNA_TRANSCRIPTION | -1.90 | 0.000 | 0.652 |
| GOBP_PROTEIN_LOCALIZATION_TO_CHROMOSOME_TELOMERIC_REGION | -1.90 | 0.002 | 0.652 |
| GOMF_GLUTATHIONE_PEROXIDASE_ACTIVITY | 1.90 | 0.000 | 0.089 |
| GOMF_EXORIBONUCLEASE_ACTIVITY | -1.90 | 0.000 | 0.662 |
| GOMF_HISTONE_KINASE_ACTIVITY | -1.90 | 0.000 | 0.667 |
| GOBP_REGULATION_OF_DNA_METABOLIC_PROCESS | -1.90 | 0.000 | 0.672 |
| GOBP_RIBONUCLEOSIDE_MONOPHOSPHATE_BIOSYNTHETIC_PROCESS | -1.90 | 0.000 | 0.683 |
| GOBP_INTRINSIC_APOPTOTIC_SIGNALING_PATHWAY_IN_RESPONSE_TO_DNA_DAMAGE_BY_P53_CLASS_MEDIATOR | 1.90 | 0.000 | 0.092 |
| GOBP_REGULATION_OF_DNA_BIOSYNTHETIC_PROCESS | -1.89 | 0.000 | 0.691 |
| GOBP_RNA_5_END_PROCESSING | -1.89 | 0.002 | 0.696 |
| GOBP_ATTACHMENT_OF_MITOTIC_SPINDLE_MICROTUBULES_TO_KINETOCHORE | -1.89 | 0.002 | 0.702 |
| GOBP_NEGATIVE_REGULATION_OF_VIRAL_PROCESS | -1.89 | 0.000 | 0.704 |
| GOBP_RNA_PHOSPHODIESTER_BOND_HYDROLYSIS_EXONUCLEOLYTIC | -1.89 | 0.000 | 0.706 |
| GOCC_ORGANELLAR_RIBOSOME | -1.89 | 0.000 | 0.713 |
| GOBP_V_D_J_RECOMBINATION | -1.89 | 0.003 | 0.730 |
| GOBP_NONRIBOSOMAL_PEPTIDE_BIOSYNTHETIC_PROCESS | 1.89 | 0.003 | 0.099 |
| GOBP_EMBRYONIC_SKELETAL_SYSTEM_DEVELOPMENT | -1.88 | 0.000 | 0.747 |
| GOMF_NEUROPEPTIDE_RECEPTOR_BINDING | -1.88 | 0.000 | 0.752 |
| GOCC_CHAPERONE_COMPLEX | -1.88 | 0.000 | 0.766 |
| GOBP_TELOMERE_MAINTENANCE | -1.88 | 0.000 | 0.777 |
| GOMF_PHOSPHATIDYLINOSITOL_3_4_BISPHOSPHATE_BINDING | 1.88 | 0.006 | 0.103 |
| GOCC_SPLICEOSOMAL_SNRNP_COMPLEX | -1.88 | 0.000 | 0.785 |
| GOBP_REGULATION_OF_TRANSLATIONAL_FIDELITY | -1.88 | 0.000 | 0.785 |
| GOBP_GLYCOSIDE_METABOLIC_PROCESS | 1.88 | 0.000 | 0.102 |
| GOBP_DNA_TEMPLATED_TRANSCRIPTION_INITIATION | -1.88 | 0.000 | 0.801 |
| GOBP_POSITIVE_REGULATION_OF_MITOTIC_CELL_CYCLE | -1.87 | 0.000 | 0.808 |
| GOBP_LYSOSOMAL_PROTEIN_CATABOLIC_PROCESS | 1.87 | 0.000 | 0.103 |
| GOMF_GLUTATHIONE_TRANSFERASE_ACTIVITY | 1.87 | 0.000 | 0.101 |
| GOBP_RRNA_METHYLATION | -1.87 | 0.000 | 0.825 |
| GOBP_ICOSANOID_BIOSYNTHETIC_PROCESS | 1.87 | 0.006 | 0.101 |
| GOMF_TRNA_BINDING | -1.87 | 0.000 | 0.841 |
| GOBP_NUCLEAR_TRANSCRIBED_MRNA_CATABOLIC_PROCESS_EXONUCLEOLYTIC | -1.86 | 0.003 | 0.855 |
| GOBP_BONE_REMODELING | 1.86 | 0.000 | 0.106 |
| GOBP_ANAPHASE_PROMOTING_COMPLEX_DEPENDENT_CATABOLIC_PROCESS | -1.86 | 0.003 | 0.874 |
| GOBP_NUCLEOSIDE_MONOPHOSPHATE_BIOSYNTHETIC_PROCESS | -1.86 | 0.000 | 0.875 |
| GOCC_KINESIN_COMPLEX | -1.86 | 0.000 | 0.880 |
| GOBP_SPLICEOSOMAL_SNRNP_ASSEMBLY | -1.86 | 0.000 | 0.881 |
| GOBP_UNSATURATED_FATTY_ACID_BIOSYNTHETIC_PROCESS | 1.86 | 0.000 | 0.109 |
| GOBP_HOMOLOGOUS_CHROMOSOME_SEGREGATION | -1.85 | 0.001 | 0.893 |
| GOBP_MITOTIC_CYTOKINESIS | -1.85 | 0.000 | 0.903 |
| GOMF_PLUS_END_DIRECTED_MICROTUBULE_MOTOR_ACTIVITY | -1.85 | 0.000 | 0.909 |
| GOCC_NUCLEAR_REPLICATION_FORK | -1.85 | 0.000 | 0.915 |
| GOBP_TYPE_B_PANCREATIC_CELL_PROLIFERATION | 1.85 | 0.003 | 0.117 |
| GOBP_OSTEOCLAST_DIFFERENTIATION | 1.85 | 0.000 | 0.115 |
| GOBP_REGULATION_OF_GENE_EXPRESSION_EPIGENETIC | -1.85 | 0.000 | 0.917 |
| GOBP_POSITIVE_REGULATION_OF_TELOMERE_MAINTENANCE | -1.84 | 0.000 | 0.923 |
| GOBP_TRNA_MODIFICATION | -1.84 | 0.000 | 0.926 |
| GOBP_MITOCHONDRIAL_GENE_EXPRESSION | -1.84 | 0.000 | 0.930 |
| GOBP_REGULATION_OF_BONE_REMODELING | 1.84 | 0.000 | 0.117 |
| GOMF_FOUR_WAY_JUNCTION_DNA_BINDING | -1.84 | 0.003 | 0.930 |
| GOBP_POSITIVE_REGULATION_OF_TELOMERE_MAINTENANCE_VIA_TELOMERE_LENGTHENING | -1.84 | 0.002 | 0.931 |
| GOBP_CYTOSKELETON_DEPENDENT_CYTOKINESIS | -1.84 | 0.000 | 0.941 |
| GOBP_INTRINSIC_APOPTOTIC_SIGNALING_PATHWAY_IN_RESPONSE_TO_DNA_DAMAGE | 1.84 | 0.000 | 0.121 |
| GOBP_POSITIVE_REGULATION_OF_MITOTIC_CELL_CYCLE_PHASE_TRANSITION | -1.84 | 0.001 | 0.942 |
| GOBP_REGULATION_OF_DOUBLE_STRAND_BREAK_REPAIR | -1.83 | 0.000 | 0.955 |
| GOBP_REGULATION_OF_NUCLEASE_ACTIVITY | -1.83 | 0.002 | 0.960 |
| GOBP_PROTEIN_CATABOLIC_PROCESS_IN_THE_VACUOLE | 1.83 | 0.003 | 0.126 |
| GOBP_PURINE_NUCLEOSIDE_MONOPHOSPHATE_BIOSYNTHETIC_PROCESS | -1.83 | 0.000 | 0.961 |
| GOBP_MRNA_MODIFICATION | -1.83 | 0.000 | 0.962 |
| GOBP_MICROGLIAL_CELL_ACTIVATION | 1.83 | 0.000 | 0.125 |
| GOBP_NCRNA_CATABOLIC_PROCESS | -1.83 | 0.000 | 0.965 |
| GOCC_SMN_SM_PROTEIN_COMPLEX | -1.83 | 0.002 | 0.965 |
| GOBP_MITOCHONDRIAL_TRANSLATION | -1.83 | 0.000 | 0.965 |
| GOBP_NEGATIVE_REGULATION_OF_DNA_RECOMBINATION | -1.82 | 0.003 | 0.967 |
| GOBP_NCRNA_3_END_PROCESSING | -1.82 | 0.002 | 0.967 |
| GOMF_RNA_POLYMERASE_ACTIVITY | -1.82 | 0.000 | 0.968 |
| GOCC_ATPASE_COMPLEX | -1.82 | 0.000 | 0.972 |
| GOBP_RESPONSE_TO_TOXIC_SUBSTANCE | 1.82 | 0.000 | 0.133 |
| GOBP_REGULATION_OF_MITOTIC_CELL_CYCLE_SPINDLE_ASSEMBLY_CHECKPOINT | -1.82 | 0.003 | 0.975 |
| GOMF_NUCLEASE_ACTIVITY | -1.82 | 0.000 | 0.975 |
| GOBP_NUCLEOLAR_LARGE_RRNA_TRANSCRIPTION_BY_RNA_POLYMERASE_I | -1.82 | 0.007 | 0.975 |
| GOBP_POSITIVE_REGULATION_OF_CELL_CYCLE_G1_S_PHASE_TRANSITION | -1.82 | 0.000 | 0.975 |
| GOBP_RNA_DEPENDENT_DNA_BIOSYNTHETIC_PROCESS | -1.81 | 0.000 | 0.976 |
| GOCC_GERM_CELL_NUCLEUS | -1.81 | 0.000 | 0.976 |
| GOMF_EXORIBONUCLEASE_ACTIVITY_PRODUCING_5_PHOSPHOMONOESTERS | -1.81 | 0.000 | 0.978 |
| GOBP_CELL_CYCLE_G2_M_PHASE_TRANSITION | -1.81 | 0.000 | 0.978 |
| GOCC_SM_LIKE_PROTEIN_FAMILY_COMPLEX | -1.81 | 0.000 | 0.979 |
| GOBP_DOUBLE_STRAND_BREAK_REPAIR_VIA_NONHOMOLOGOUS_END_JOINING | -1.81 | 0.000 | 0.980 |
| GOMF_DNA_SECONDARY_STRUCTURE_BINDING | -1.81 | 0.000 | 0.983 |
| GOBP_HISTONE_METHYLATION | -1.81 | 0.000 | 0.984 |
| GOBP_CELL_REDOX_HOMEOSTASIS | 1.80 | 0.000 | 0.149 |
| GOBP_TRANSLATIONAL_INITIATION | -1.80 | 0.000 | 0.986 |
| GOCC_RIBOSOME | -1.80 | 0.000 | 0.986 |
| GOBP_POSITIVE_REGULATION_OF_TELOMERASE_RNA_LOCALIZATION_TO_CAJAL_BODY | -1.80 | 0.000 | 0.986 |
| GOMF_LYSOPHOSPHOLIPASE_ACTIVITY | 1.80 | 0.005 | 0.149 |
| GOBP_RRNA_CATABOLIC_PROCESS | -1.80 | 0.003 | 0.990 |
| GOBP_AMINO_ACID_ACTIVATION | -1.80 | 0.000 | 0.990 |
| GOBP_POSITIVE_REGULATION_OF_BONE_RESORPTION | 1.80 | 0.011 | 0.150 |
| GOBP_UNSATURATED_FATTY_ACID_METABOLIC_PROCESS | 1.80 | 0.000 | 0.151 |
| GOBP_PLASMA_LIPOPROTEIN_PARTICLE_CLEARANCE | 1.79 | 0.003 | 0.149 |
| GOBP_PYRIMIDINE_NUCLEOTIDE_METABOLIC_PROCESS | -1.79 | 0.000 | 0.991 |
| GOBP_REGULATION_OF_OSTEOCLAST_DIFFERENTIATION | 1.79 | 0.000 | 0.148 |
| GOCC_PHAGOPHORE_ASSEMBLY_SITE_MEMBRANE | 1.79 | 0.008 | 0.148 |
| GOBP_MEIOSIS_I_CELL_CYCLE_PROCESS | -1.79 | 0.000 | 0.991 |
| GOCC_MITOCHONDRIAL_LARGE_RIBOSOMAL_SUBUNIT | -1.79 | 0.000 | 0.991 |
| GOBP_NEGATIVE_REGULATION_OF_NUCLEOTIDE_METABOLIC_PROCESS | 1.79 | 0.005 | 0.149 |
| GOBP_ESTABLISHMENT_OF_PROTEIN_LOCALIZATION_TO_TELOMERE | -1.79 | 0.000 | 0.992 |
| GOBP_NEGATIVE_REGULATION_OF_MITOTIC_CELL_CYCLE | -1.79 | 0.000 | 0.992 |
| GOBP_PYRIMIDINE_DEOXYRIBONUCLEOTIDE_METABOLIC_PROCESS | -1.78 | 0.004 | 0.994 |
| GOBP_EMBRYONIC_DIGESTIVE_TRACT_MORPHOGENESIS | -1.78 | 0.003 | 0.994 |
| GOMF_METAL_CLUSTER_BINDING | -1.78 | 0.000 | 0.994 |
| GOBP_NEGATIVE_REGULATION_OF_MRNA_METABOLIC_PROCESS | -1.78 | 0.001 | 0.995 |
| GOBP_POSITIVE_REGULATION_OF_DNA_METABOLIC_PROCESS | -1.78 | 0.000 | 0.995 |
| GOBP_CARDIAC_CONDUCTION_SYSTEM_DEVELOPMENT | -1.78 | 0.000 | 0.995 |
| GOBP_AUTOPHAGY_OF_MITOCHONDRION | 1.78 | 0.000 | 0.157 |
| GOMF_4_IRON_4_SULFUR_CLUSTER_BINDING | -1.78 | 0.000 | 0.996 |
| GOBP_NEGATIVE_REGULATION_OF_LIPASE_ACTIVITY | 1.78 | 0.008 | 0.157 |
| GOMF_S_ADENOSYLMETHIONINE_DEPENDENT_METHYLTRANSFERASE_ACTIVITY | -1.78 | 0.000 | 0.997 |
| GOMF_DNA_BINDING_BENDING | -1.78 | 0.002 | 0.997 |
| GOBP_NEURON_FATE_SPECIFICATION | -1.78 | 0.002 | 0.997 |
| GOBP_INTRINSIC_APOPTOTIC_SIGNALING_PATHWAY_BY_P53_CLASS_MEDIATOR | 1.78 | 0.000 | 0.158 |
| GOCC_VACUOLAR_MEMBRANE | 1.78 | 0.000 | 0.156 |
| GOBP_INTERSTRAND_CROSS_LINK_REPAIR | -1.78 | 0.000 | 0.997 |
| GOCC_METHYLTRANSFERASE_COMPLEX | -1.77 | 0.000 | 0.998 |
| GOBP_POSITIVE_REGULATION_OF_TRANSCRIPTION_BY_RNA_POLYMERASE_I | -1.77 | 0.005 | 0.998 |
| GOBP_ZINC_ION_HOMEOSTASIS | -1.77 | 0.001 | 0.998 |
| GOMF_HISTONE_BINDING | -1.77 | 0.000 | 0.998 |
| GOMF_TRANSFERASE_ACTIVITY_TRANSFERRING_ONE_CARBON_GROUPS | -1.77 | 0.000 | 0.998 |
| GOBP_BROWN_FAT_CELL_DIFFERENTIATION | 1.77 | 0.000 | 0.167 |
| GOCC_LAMELLAR_BODY | 1.77 | 0.010 | 0.165 |
| GOCC_EARLY_ENDOSOME_MEMBRANE | 1.77 | 0.000 | 0.163 |
| GOBP_NUCLEOLUS_ORGANIZATION | -1.77 | 0.006 | 0.998 |
| GOMF_DOUBLE_STRANDED_RNA_BINDING | -1.77 | 0.000 | 0.998 |
| GOBP_TELOMERE_MAINTENANCE_VIA_TELOMERE_LENGTHENING | -1.77 | 0.000 | 0.998 |
| GOBP_POSITIVE_REGULATION_OF_HISTONE_METHYLATION | -1.76 | 0.005 | 0.998 |
| GOCC_U5_SNRNP | -1.76 | 0.002 | 0.998 |
| GOBP_CENTRIOLE_ASSEMBLY | -1.76 | 0.003 | 0.998 |
| GOCC_NUCLEAR_UBIQUITIN_LIGASE_COMPLEX | -1.76 | 0.001 | 0.998 |
| GOMF_LIGASE_ACTIVITY_FORMING_CARBON_OXYGEN_BONDS | -1.76 | 0.001 | 0.998 |
| GOCC_RIBOSOMAL_SUBUNIT | -1.76 | 0.000 | 0.998 |
| GOBP_ICOSANOID_METABOLIC_PROCESS | 1.76 | 0.000 | 0.169 |
| GOBP_GROOMING_BEHAVIOR | -1.76 | 0.005 | 0.998 |
| GOBP_NUCLEAR_EXPORT | -1.76 | 0.000 | 0.998 |
| GOMF_SNRNA_BINDING | -1.76 | 0.004 | 0.998 |
| GOBP_POSITIVE_REGULATION_OF_AUTOPHAGY | 1.76 | 0.000 | 0.171 |
| GOBP_REGULATION_OF_TRANSCRIPTION_BY_RNA_POLYMERASE_I | -1.76 | 0.002 | 0.998 |
| GOBP_ANTIVIRAL_INNATE_IMMUNE_RESPONSE | -1.75 | 0.005 | 0.998 |
| GOCC_ENDOSOME_MEMBRANE | 1.75 | 0.000 | 0.173 |
| GOBP_INTERMEDIATE_FILAMENT_BASED_PROCESS | 1.75 | 0.003 | 0.172 |
| GOBP_HISTONE_PHOSPHORYLATION | -1.75 | 0.003 | 0.998 |
| GOBP_VACUOLE_ORGANIZATION | 1.75 | 0.000 | 0.172 |
| GOBP_POSITIVE_REGULATION_OF_RESPONSE_TO_DNA_DAMAGE_STIMULUS | -1.75 | 0.000 | 0.998 |
| GOCC_AXONEMAL_DYNEIN_COMPLEX | 1.75 | 0.006 | 0.174 |
| GOBP_MICROTUBULE_NUCLEATION | -1.75 | 0.000 | 0.998 |
| GOBP_REGULATION_OF_EXIT_FROM_MITOSIS | -1.75 | 0.005 | 0.998 |
| GOBP_PROTEIN_TRANSMEMBRANE_TRANSPORT | -1.75 | 0.000 | 0.998 |
| GOBP_RESPONSE_TO_ELECTRICAL_STIMULUS | 1.74 | 0.000 | 0.177 |
| GOBP_URETER_DEVELOPMENT | -1.74 | 0.008 | 0.998 |
| GOCC_MRNA_CLEAVAGE_FACTOR_COMPLEX | -1.74 | 0.009 | 0.998 |
| GOCC_GOLGI_CIS_CISTERNA | 1.74 | 0.008 | 0.177 |
| GOBP_REGULATION_OF_DNA_RECOMBINATION | -1.74 | 0.000 | 0.998 |
| GOMF_PROTEIN_FOLDING_CHAPERONE | -1.74 | 0.005 | 0.998 |
| GOBP_CELLULAR_IRON_ION_HOMEOSTASIS | 1.74 | 0.000 | 0.177 |
| GOMF_RRNA_BINDING | -1.74 | 0.000 | 0.998 |
| GOCC_GLIAL_CELL_PROJECTION | 1.74 | 0.011 | 0.176 |
| GOBP_NUCLEUS_ORGANIZATION | -1.74 | 0.000 | 0.998 |
| GOBP_REGULATION_OF_CELL_CYCLE_G2_M_PHASE_TRANSITION | -1.74 | 0.000 | 0.998 |
| GOBP_POSITIVE_REGULATION_OF_DOUBLE_STRAND_BREAK_REPAIR | -1.74 | 0.000 | 0.998 |
| GOMF_CHROMATIN_DNA_BINDING | -1.73 | 0.000 | 0.998 |
| GOBP_NEURAL_CREST_FORMATION | -1.73 | 0.008 | 0.998 |
| GOBP_METHYLATION | -1.73 | 0.000 | 0.998 |
| GOBP_PEPTIDYL_LYSINE_METHYLATION | -1.73 | 0.000 | 0.998 |
| GOBP_AXONAL_TRANSPORT_OF_MITOCHONDRION | 1.73 | 0.010 | 0.191 |
| GOBP_POSITIVE_REGULATION_OF_DNA_REPAIR | -1.73 | 0.001 | 0.998 |
| GOBP_CERAMIDE_CATABOLIC_PROCESS | 1.73 | 0.016 | 0.189 |
| GOBP_SKELETAL_MUSCLE_CELL_DIFFERENTIATION | 1.73 | 0.003 | 0.189 |
| GOBP_NEGATIVE_REGULATION_OF_HISTONE_MODIFICATION | -1.73 | 0.004 | 0.998 |
| GOBP_REGULATION_OF_MRNA_METABOLIC_PROCESS | -1.72 | 0.000 | 0.998 |
| GOMF_RIBONUCLEASE_ACTIVITY | -1.72 | 0.000 | 0.998 |
| GOCC_FIBRILLAR_CENTER | -1.72 | 0.000 | 0.998 |
| GOMF_DAMAGED_DNA_BINDING | -1.72 | 0.001 | 0.998 |
| GOMF_NUCLEOTIDYLTRANSFERASE_ACTIVITY | -1.72 | 0.001 | 0.998 |
| GOBP_VIRAL_GENOME_REPLICATION | -1.72 | 0.000 | 0.998 |
| GOMF_OXIDOREDUCTASE_ACTIVITY_ACTING_ON_THE_CH_CH_GROUP_OF_DONORS_NAD_OR_NADP_AS_ACCEPTOR | 1.72 | 0.018 | 0.196 |
| GOCC_ASTROCYTE_PROJECTION | 1.72 | 0.007 | 0.196 |
| GOBP_VASCULAR_WOUND_HEALING | -1.72 | 0.008 | 0.999 |
| GOBP_DNA_TEMPLATED_TRANSCRIPTION_TERMINATION | -1.72 | 0.013 | 0.999 |
| GOMF_ALCOHOL_DEHYDROGENASE_NADPPLUS_ACTIVITY | 1.72 | 0.016 | 0.198 |
| GOBP_SKELETAL_SYSTEM_MORPHOGENESIS | -1.71 | 0.000 | 0.999 |
| GOBP_NEGATIVE_REGULATION_OF_DEVELOPMENTAL_GROWTH | 1.71 | 0.000 | 0.197 |
| GOMF_RIBONUCLEOPROTEIN_COMPLEX_BINDING | -1.71 | 0.000 | 0.999 |
| GOBP_PEPTIDYL_LYSINE_TRIMETHYLATION | -1.71 | 0.004 | 0.999 |
| GOCC_SYNAPTONEMAL_STRUCTURE | -1.71 | 0.006 | 0.999 |
| GOBP_TRANSCRIPTION_BY_RNA_POLYMERASE_I | -1.71 | 0.001 | 0.999 |
| GOBP_POSITIVE_REGULATION_OF_RHO_PROTEIN_SIGNAL_TRANSDUCTION | 1.71 | 0.011 | 0.207 |
| GOMF_MICROTUBULE_MOTOR_ACTIVITY | -1.71 | 0.003 | 1.000 |
| GOBP_CENTROSOME_DUPLICATION | -1.71 | 0.001 | 1.000 |
| GOBP_NEGATIVE_REGULATION_OF_HISTONE_METHYLATION | -1.70 | 0.013 | 1.000 |
| GOBP_HISTONE_MODIFICATION | -1.70 | 0.000 | 1.000 |
| GOCC_LARGE_RIBOSOMAL_SUBUNIT | -1.70 | 0.000 | 1.000 |
| GOBP_EMBRYONIC_ORGAN_MORPHOGENESIS | -1.70 | 0.000 | 1.000 |
| GOBP_REGULATION_OF_HISTONE_METHYLATION | -1.70 | 0.000 | 1.000 |
| GOBP_MICROTUBULE_BASED_TRANSPORT | 1.70 | 0.000 | 0.214 |
| GOCC_EUCHROMATIN | -1.70 | 0.002 | 1.000 |
| GOBP_SNRNA_PROCESSING | -1.70 | 0.011 | 1.000 |
| GOCC_CELL_DIVISION_SITE | -1.70 | 0.003 | 1.000 |
| GOBP_AUTOPHAGOSOME_MATURATION | 1.70 | 0.003 | 0.215 |
| GOBP_REGULATION_OF_DNA_METHYLATION | -1.70 | 0.007 | 1.000 |
| GOBP_NUCLEOSIDE_MONOPHOSPHATE_METABOLIC_PROCESS | -1.70 | 0.000 | 1.000 |
| GOBP_MITOTIC_SISTER_CHROMATID_COHESION | -1.69 | 0.011 | 1.000 |
| GOBP_CELLULAR_RESPONSE_TO_CHEMICAL_STRESS | 1.69 | 0.000 | 0.220 |
| GOBP_POSITIVE_REGULATION_OF_NATURAL_KILLER_CELL_ACTIVATION | 1.69 | 0.009 | 0.220 |
| GOMF_MAGNESIUM_ION_TRANSMEMBRANE_TRANSPORTER_ACTIVITY | 1.69 | 0.018 | 0.217 |
| GOBP_NEGATIVE_REGULATION_OF_NIK_NF_KAPPAB_SIGNALING | 1.69 | 0.020 | 0.215 |
| GOBP_REGULATION_OF_CELL_DIVISION | -1.69 | 0.000 | 1.000 |
| GOBP_POSITIVE_REGULATION_OF_HISTONE_MODIFICATION | -1.69 | 0.000 | 1.000 |
| GOCC_ANAPHASE_PROMOTING_COMPLEX | -1.69 | 0.014 | 1.000 |
| GOBP_RNA_3_END_PROCESSING | -1.69 | 0.000 | 1.000 |
| GOBP_CELLULAR_RESPONSE_TO_ZINC_ION | -1.69 | 0.011 | 1.000 |
| GOCC_LATE_ENDOSOME | 1.69 | 0.000 | 0.222 |
| GOBP_NEGATIVE_REGULATION_OF_STRIATED_MUSCLE_CELL_APOPTOTIC_PROCESS | 1.69 | 0.006 | 0.220 |
| GOMF_HORMONE_RECEPTOR_BINDING | -1.69 | 0.011 | 1.000 |
| GOBP_REGULATION_OF_EPITHELIAL_CELL_APOPTOTIC_PROCESS | 1.69 | 0.004 | 0.219 |
| GOBP_MICROTUBULE_ORGANIZING_CENTER_ORGANIZATION | -1.69 | 0.001 | 1.000 |
| GOBP_MAGNESIUM_ION_TRANSPORT | 1.69 | 0.016 | 0.217 |
| GOBP_ANGIOGENESIS_INVOLVED_IN_WOUND_HEALING | -1.69 | 0.013 | 1.000 |
| GOBP_BASE_EXCISION_REPAIR | -1.68 | 0.002 | 1.000 |
| GOBP_RESPONSE_TO_ISCHEMIA | 1.68 | 0.000 | 0.219 |
| GOMF_MRNA_BINDING | -1.68 | 0.000 | 1.000 |
| GOBP_DNA_INTEGRITY_CHECKPOINT_SIGNALING | -1.68 | 0.000 | 1.000 |
| GOBP_STEROL_BIOSYNTHETIC_PROCESS | 1.68 | 0.000 | 0.218 |
| GOMF_TELOMERIC_DNA_BINDING | -1.68 | 0.006 | 1.000 |
| GOMF_RETINAL_BINDING | -1.68 | 0.006 | 1.000 |
| GOMF_CALMODULIN_DEPENDENT_PROTEIN_KINASE_ACTIVITY | -1.68 | 0.008 | 1.000 |
| GOBP_REGULATION_OF_TRANSCRIPTION_OF_NUCLEOLAR_LARGE_RRNA_BY_RNA_POLYMERASE_I | -1.68 | 0.008 | 1.000 |
| GOBP_NEUROINFLAMMATORY_RESPONSE | 1.68 | 0.012 | 0.219 |
| GOCC_MRNA_CLEAVAGE_AND_POLYADENYLATION_SPECIFICITY_FACTOR_COMPLEX | -1.68 | 0.009 | 1.000 |
| GOCC_U12_TYPE_SPLICEOSOMAL_COMPLEX | -1.68 | 0.008 | 1.000 |
| GOBP_TRNA_METHYLATION | -1.68 | 0.003 | 1.000 |
| GOCC_DNA_REPAIR_COMPLEX | -1.68 | 0.006 | 1.000 |
| GOBP_G1_TO_G0_TRANSITION | -1.68 | 0.013 | 1.000 |
| GOBP_LEFT_RIGHT_PATTERN_FORMATION | 1.68 | 0.015 | 0.225 |
| GOBP_MITOCHONDRIAL_RNA_METABOLIC_PROCESS | -1.68 | 0.001 | 1.000 |
| GOBP_POSITIVE_REGULATION_OF_CELL_CYCLE_G2_M_PHASE_TRANSITION | -1.67 | 0.008 | 1.000 |
| GOBP_NEGATIVE_REGULATION_OF_VIRAL_LIFE_CYCLE | -1.67 | 0.008 | 1.000 |
| GOBP_MITOCHONDRIAL_TRANSMEMBRANE_TRANSPORT | -1.67 | 0.006 | 1.000 |
| GOBP_REGULATION_OF_SISTER_CHROMATID_COHESION | -1.67 | 0.010 | 1.000 |
| GOBP_PSEUDOURIDINE_SYNTHESIS | -1.67 | 0.006 | 1.000 |
| GOBP_MEIOTIC_CHROMOSOME_SEPARATION | -1.67 | 0.011 | 1.000 |
| GOMF_TRNA_METHYLTRANSFERASE_ACTIVITY | -1.67 | 0.005 | 1.000 |
| GOBP_NUCLEOBASE_CONTAINING_COMPOUND_TRANSPORT | -1.67 | 0.000 | 1.000 |
| GOBP_REGULATION_OF_DNA_REPAIR | -1.67 | 0.001 | 1.000 |
| GOBP_PROTEIN_LOCALIZATION_TO_CHROMATIN | -1.67 | 0.003 | 1.000 |
| GOBP_NEGATIVE_REGULATION_OF_HISTONE_ACETYLATION | -1.67 | 0.014 | 1.000 |
| GOMF_UNFOLDED_PROTEIN_BINDING | -1.67 | 0.000 | 1.000 |
| GOBP_POSITIVE_REGULATION_OF_MRNA_SPLICING_VIA_SPLICEOSOME | -1.67 | 0.006 | 1.000 |
| GOBP_CEREBELLAR_PURKINJE_CELL_LAYER_DEVELOPMENT | -1.67 | 0.006 | 1.000 |
| GOCC_RESPIRATORY_CHAIN_COMPLEX_IV | 1.67 | 0.031 | 0.239 |
| GOBP_REPLICATION_FORK_PROCESSING | -1.67 | 0.004 | 1.000 |
| GOBP_ANTERIOR_POSTERIOR_PATTERN_SPECIFICATION | -1.67 | 0.000 | 1.000 |
| GOBP_REGULATION_OF_CYCLIN_DEPENDENT_PROTEIN_KINASE_ACTIVITY | -1.66 | 0.000 | 1.000 |
| GOBP_AXO_DENDRITIC_TRANSPORT | 1.66 | 0.008 | 0.243 |
| GOCC_REPLISOME | -1.66 | 0.008 | 1.000 |
| GOCC_CIS_GOLGI_NETWORK | 1.66 | 0.003 | 0.241 |
| GOBP_CELLULAR_RESPONSE_TO_PROSTAGLANDIN_STIMULUS | 1.66 | 0.021 | 0.239 |
| GOMF_PROTEIN_KINASE_C_BINDING | 1.66 | 0.000 | 0.237 |
| GOBP_REGULATION_OF_PHOSPHATIDYLINOSITOL_3_KINASE_ACTIVITY | -1.66 | 0.009 | 1.000 |
| GOBP_PIGMENT_GRANULE_ORGANIZATION | 1.66 | 0.016 | 0.235 |
| GOBP_CELL_CYCLE_G1_S_PHASE_TRANSITION | -1.66 | 0.001 | 1.000 |
| GOBP_RESPONSE_TO_OXYGEN_RADICAL | 1.66 | 0.029 | 0.236 |
| GOBP_REGULATION_OF_HISTONE_MODIFICATION | -1.66 | 0.000 | 1.000 |
| GOBP_POSITIVE_REGULATION_OF_MITOTIC_NUCLEAR_DIVISION | -1.66 | 0.005 | 1.000 |
| GOBP_MRNA_CLEAVAGE | -1.66 | 0.019 | 1.000 |
| GOBP_MEMBRANE_LIPID_CATABOLIC_PROCESS | 1.65 | 0.015 | 0.244 |
| GOBP_REGULATION_OF_MEIOTIC_CELL_CYCLE | -1.65 | 0.003 | 1.000 |
| GOBP_RNA_CATABOLIC_PROCESS | -1.65 | 0.000 | 1.000 |
| GOMF_ENDORIBONUCLEASE_ACTIVITY_PRODUCING_5_PHOSPHOMONOESTERS | -1.65 | 0.011 | 1.000 |
| GOBP_AMP_METABOLIC_PROCESS | -1.65 | 0.012 | 1.000 |
| GOBP_EMBRYONIC_ORGAN_DEVELOPMENT | -1.65 | 0.000 | 1.000 |
| GOBP_EPITHELIAL_TUBE_BRANCHING_INVOLVED_IN_LUNG_MORPHOGENESIS | -1.65 | 0.014 | 1.000 |
| GOMF_STRUCTURAL_CONSTITUENT_OF_RIBOSOME | -1.65 | 0.001 | 1.000 |
| GOBP_ALTERNATIVE_MRNA_SPLICING_VIA_SPLICEOSOME | -1.65 | 0.004 | 1.000 |
| GOMF_PROTEIN_SERINE_THREONINE_KINASE_ACTIVATOR_ACTIVITY | -1.65 | 0.006 | 1.000 |
| GOBP_REGULATION_OF_SPINDLE_ORGANIZATION | -1.65 | 0.013 | 1.000 |
| GOBP_CHEMOKINE_PRODUCTION | 1.65 | 0.004 | 0.255 |
| GOBP_CALCIUM_DEPENDENT_CELL_CELL_ADHESION_VIA_PLASMA_MEMBRANE_CELL_ADHESION_MOLECULES | 1.65 | 0.008 | 0.255 |
| GOMF_N_METHYLTRANSFERASE_ACTIVITY | -1.65 | 0.001 | 1.000 |
| GOBP_INTRACELLULAR_PROTEIN_TRANSMEMBRANE_TRANSPORT | -1.64 | 0.001 | 1.000 |
| GOBP_REGULATION_OF_SUPEROXIDE_METABOLIC_PROCESS | 1.64 | 0.012 | 0.257 |
| GOBP_TRANSPORT_ALONG_MICROTUBULE | 1.64 | 0.000 | 0.256 |
| GOBP_OLIGOSACCHARIDE_LIPID_INTERMEDIATE_BIOSYNTHETIC_PROCESS | -1.64 | 0.016 | 1.000 |
| GOBP_VIRAL_GENE_EXPRESSION | -1.64 | 0.000 | 1.000 |
| GOMF_P53_BINDING | -1.64 | 0.009 | 1.000 |
| GOBP_POSITIVE_REGULATION_OF_ATP_DEPENDENT_ACTIVITY | -1.64 | 0.006 | 1.000 |
| GOBP_POSITIVE_REGULATION_OF_TRANSCRIPTION_FROM_RNA_POLYMERASE_II_PROMOTER_IN_RESPONSE_TO_STRESS | 1.64 | 0.014 | 0.256 |
| GOBP_PROTEIN_DNA_COMPLEX_DISASSEMBLY | -1.64 | 0.018 | 1.000 |
| GOCC_APICAL_DENDRITE | 1.64 | 0.025 | 0.258 |
| GOMF_RETINOL_BINDING | -1.64 | 0.010 | 1.000 |
| GOBP_NEGATIVE_REGULATION_OF_PEPTIDYL_LYSINE_ACETYLATION | -1.64 | 0.011 | 1.000 |
| GOBP_NEGATIVE_REGULATION_OF_AXON_EXTENSION | 1.64 | 0.009 | 0.260 |
| GOCC_RNA_POLYMERASE_COMPLEX | -1.64 | 0.000 | 1.000 |
| GOBP_INNATE_IMMUNE_RESPONSE_IN_MUCOSA | -1.64 | 0.018 | 1.000 |
| GOCC_TRICARBOXYLIC_ACID_CYCLE_ENZYME_COMPLEX | -1.64 | 0.007 | 1.000 |
| GOBP_QUINONE_METABOLIC_PROCESS | 1.63 | 0.015 | 0.263 |
| GOCC_SITE_OF_DNA_DAMAGE | -1.63 | 0.003 | 1.000 |
| GOBP_LATE_ENDOSOME_TO_LYSOSOME_TRANSPORT | 1.63 | 0.029 | 0.263 |
| GOBP_NEGATIVE_REGULATION_OF_MACROPHAGE_ACTIVATION | 1.63 | 0.019 | 0.261 |
| GOBP_ANATOMICAL_STRUCTURE_REGRESSION | -1.63 | 0.020 | 1.000 |
| GOBP_REGULATION_OF_HISTONE_H3_K9_METHYLATION | -1.63 | 0.017 | 1.000 |
| GOCC_VOLTAGE_GATED_SODIUM_CHANNEL_COMPLEX | 1.63 | 0.018 | 0.263 |
| GOBP_INTRINSIC_APOPTOTIC_SIGNALING_PATHWAY | 1.63 | 0.000 | 0.261 |
| GOBP_PYRIMIDINE_NUCLEOTIDE_CATABOLIC_PROCESS | -1.63 | 0.017 | 1.000 |
| GOBP_REGULATION_OF_TRANSCRIPTION_REGULATORY_REGION_DNA_BINDING | -1.63 | 0.003 | 1.000 |
| GOMF_NEUROPEPTIDE_HORMONE_ACTIVITY | -1.63 | 0.018 | 1.000 |
| GOBP_SNRNA_METABOLIC_PROCESS | -1.63 | 0.002 | 1.000 |
| GOBP_HISTONE_H3_K27_METHYLATION | -1.63 | 0.010 | 1.000 |
| GOBP_NEGATIVE_REGULATION_OF_PROTEIN_ACETYLATION | -1.63 | 0.018 | 1.000 |
| GOBP_OLIGODENDROCYTE_DIFFERENTIATION | -1.63 | 0.001 | 1.000 |
| GOBP_IMMUNOLOGICAL_MEMORY_PROCESS | 1.63 | 0.019 | 0.266 |
| GOBP_MHC_CLASS_II_BIOSYNTHETIC_PROCESS | -1.63 | 0.017 | 1.000 |
| GOBP_NEGATIVE_REGULATION_OF_SMALL_MOLECULE_METABOLIC_PROCESS | 1.63 | 0.007 | 0.265 |
| GOBP_ESTABLISHMENT_OF_PIGMENT_GRANULE_LOCALIZATION | 1.63 | 0.032 | 0.263 |
| GOBP_DNA_DAMAGE_RESPONSE_SIGNAL_TRANSDUCTION_BY_P53_CLASS_MEDIATOR_RESULTING_IN_CELL_CYCLE_ARREST | 1.63 | 0.028 | 0.262 |
| GOBP_NEGATIVE_REGULATION_OF_NEURAL_PRECURSOR_CELL_PROLIFERATION | 1.63 | 0.034 | 0.260 |
| GOBP_PROTEIN_FOLDING | -1.62 | 0.000 | 1.000 |
| GOBP_FAT_CELL_DIFFERENTIATION | 1.62 | 0.000 | 0.260 |
| GOBP_POSITIVE_REGULATION_OF_CHEMOKINE_PRODUCTION | 1.62 | 0.000 | 0.258 |
| GOBP_HISTONE_MRNA_METABOLIC_PROCESS | -1.62 | 0.015 | 1.000 |
| GOBP_HINDLIMB_MORPHOGENESIS | -1.62 | 0.009 | 1.000 |
| GOBP_PH_REDUCTION | 1.62 | 0.019 | 0.258 |
| GOCC_MALE_GERM_CELL_NUCLEUS | -1.62 | 0.023 | 1.000 |
| GOBP_PROTEIN_METHYLATION | -1.62 | 0.000 | 1.000 |
| GOBP_COLLECTING_DUCT_DEVELOPMENT | 1.62 | 0.033 | 0.257 |
| GOMF_TRANSLATION_FACTOR_ACTIVITY_RNA_BINDING | -1.62 | 0.004 | 1.000 |
| GOBP_REGULATION_OF_VIRAL_PROCESS | -1.62 | 0.001 | 1.000 |
| GOBP_POSITIVE_REGULATION_OF_DOUBLE_STRAND_BREAK_REPAIR_VIA_NONHOMOLOGOUS_END_JOINING | -1.62 | 0.016 | 1.000 |
| GOMF_GLUCOSYLTRANSFERASE_ACTIVITY | -1.62 | 0.024 | 1.000 |
| GOBP_PYRIMIDINE_NUCLEOSIDE_TRIPHOSPHATE_METABOLIC_PROCESS | -1.62 | 0.016 | 1.000 |
| GOBP_EMBRYONIC_DIGESTIVE_TRACT_DEVELOPMENT | -1.62 | 0.013 | 1.000 |
| GOBP_STEROID_BIOSYNTHETIC_PROCESS | 1.62 | 0.000 | 0.261 |
| GOBP_SOMATIC_DIVERSIFICATION_OF_IMMUNE_RECEPTORS | -1.62 | 0.003 | 1.000 |
| GOBP_REGULATION_OF_CHROMATIN_BINDING | -1.62 | 0.018 | 1.000 |
| GOBP_CELLULAR_RESPONSE_TO_EXTERNAL_STIMULUS | 1.62 | 0.000 | 0.262 |
| GOBP_EXIT_FROM_MITOSIS | -1.62 | 0.014 | 1.000 |
| GOBP_PROTEIN_TRANSMEMBRANE_IMPORT_INTO_INTRACELLULAR_ORGANELLE | -1.62 | 0.009 | 1.000 |
| GOBP_NUCLEAR_TRANSPORT | -1.62 | 0.000 | 1.000 |
| GOBP_LYSOSOMAL_TRANSPORT | 1.61 | 0.004 | 0.266 |
| GOBP_RIBONUCLEOSIDE_MONOPHOSPHATE_METABOLIC_PROCESS | -1.61 | 0.003 | 1.000 |
| GOBP_REGULATION_OF_DOUBLE_STRAND_BREAK_REPAIR_VIA_NONHOMOLOGOUS_END_JOINING | -1.61 | 0.016 | 1.000 |
| GOBP_LACTATION | -1.61 | 0.019 | 1.000 |
| GOCC_MITOCHONDRIAL_MATRIX | -1.61 | 0.000 | 1.000 |
| GOBP_NEGATIVE_REGULATION_OF_STRIATED_MUSCLE_CELL_DIFFERENTIATION | -1.61 | 0.009 | 1.000 |
| GOBP_NEGATIVE_REGULATION_OF_TELOMERE_MAINTENANCE | -1.61 | 0.011 | 1.000 |
| GOBP_MITOCHONDRIAL_RNA_PROCESSING | -1.61 | 0.018 | 1.000 |
| GOCC_NUCLEAR_SPECK | -1.61 | 0.000 | 1.000 |
| GOBP_PHOSPHATE_ION_TRANSMEMBRANE_TRANSPORT | -1.61 | 0.028 | 1.000 |
| GOMF_PHOSPHATIDYLINOSITOL_3_KINASE_REGULATOR_ACTIVITY | -1.61 | 0.015 | 1.000 |
| GOMF_TELOMERASE_RNA_BINDING | -1.61 | 0.027 | 1.000 |
| GOBP_EMBRYONIC_EYE_MORPHOGENESIS | -1.61 | 0.014 | 1.000 |
| GOBP_SPLICEOSOMAL_COMPLEX_ASSEMBLY | -1.61 | 0.007 | 1.000 |
| GOBP_REGULATION_OF_GLIAL_CELL_MIGRATION | 1.61 | 0.037 | 0.279 |
| GOBP_NUCLEOBASE_METABOLIC_PROCESS | -1.61 | 0.007 | 1.000 |
| GOMF_LONG_CHAIN_FATTY_ACID_TRANSPORTER_ACTIVITY | -1.61 | 0.021 | 1.000 |
| GOCC_NUCLEAR_PORE | -1.61 | 0.003 | 1.000 |
| GOMF_ENDONUCLEASE_ACTIVITY_ACTIVE_WITH_EITHER_RIBO_OR_DEOXYRIBONUCLEIC_ACIDS_AND_PRODUCING_5_PHOSPHOMONOESTERS | -1.61 | 0.013 | 1.000 |
| GOMF_BHLH_TRANSCRIPTION_FACTOR_BINDING | -1.61 | 0.017 | 1.000 |
| GOBP_IMP_METABOLIC_PROCESS | -1.61 | 0.023 | 1.000 |
| GOBP_AXONAL_TRANSPORT | 1.60 | 0.006 | 0.282 |
| GOCC_CENTRIOLE | -1.60 | 0.001 | 1.000 |
| GOCC_U2_TYPE_CATALYTIC_STEP_2_SPLICEOSOME | -1.60 | 0.014 | 1.000 |
| GOBP_IRON_ION_TRANSPORT | 1.60 | 0.009 | 0.281 |
| GOBP_CELLULAR_RESPONSE_TO_STARVATION | 1.60 | 0.000 | 0.281 |
| GOBP_RESPONSE_TO_COCAINE | -1.60 | 0.016 | 1.000 |
| GOCC_CLEAVAGE_FURROW | -1.60 | 0.012 | 1.000 |
| GOBP_MICROTUBULE_POLYMERIZATION_OR_DEPOLYMERIZATION | -1.60 | 0.003 | 1.000 |
| GOBP_NEGATIVE_REGULATION_OF_INTRINSIC_APOPTOTIC_SIGNALING_PATHWAY_IN_RESPONSE_TO_DNA_DAMAGE | 1.60 | 0.020 | 0.281 |
| GOCC_U2_SNRNP | -1.60 | 0.024 | 1.000 |
| GOCC_PERICENTRIC_HETEROCHROMATIN | -1.60 | 0.023 | 1.000 |
| GOBP_POSITIVE_REGULATION_OF_RNA_SPLICING | -1.60 | 0.015 | 1.000 |
| GOBP_INTERLEUKIN_6_MEDIATED_SIGNALING_PATHWAY | 1.60 | 0.039 | 0.281 |
| GOBP_TRNA_5_END_PROCESSING | -1.60 | 0.015 | 1.000 |
| GOMF_SULFUR_COMPOUND_TRANSMEMBRANE_TRANSPORTER_ACTIVITY | 1.60 | 0.006 | 0.280 |
| GOBP_EMBRYONIC_APPENDAGE_MORPHOGENESIS | -1.60 | 0.003 | 1.000 |
| GOBP_BLASTOCYST_GROWTH | -1.60 | 0.013 | 1.000 |
| GOCC_ENDORIBONUCLEASE_COMPLEX | -1.60 | 0.014 | 1.000 |
| GOBP_POSITIVE_REGULATION_OF_TRANSLATIONAL_INITIATION | -1.60 | 0.014 | 1.000 |
| GOBP_IRON_ION_HOMEOSTASIS | 1.60 | 0.003 | 0.284 |
| GOBP_INTERLEUKIN_5_PRODUCTION | -1.60 | 0.029 | 1.000 |
| GOCC_PRESPLICEOSOME | -1.60 | 0.027 | 1.000 |
| GOBP_CELLULAR_RESPONSE_TO_REACTIVE_NITROGEN_SPECIES | -1.59 | 0.013 | 1.000 |
| GOBP_TISSUE_REMODELING | 1.59 | 0.000 | 0.287 |
| GOBP_EPIDERMIS_MORPHOGENESIS | 1.59 | 0.030 | 0.288 |
| GOBP_NUCLEAR_TRANSCRIBED_MRNA_CATABOLIC_PROCESS_NONSENSE_MEDIATED_DECAY | -1.59 | 0.006 | 1.000 |
| GOBP_PROGESTERONE_METABOLIC_PROCESS | 1.59 | 0.044 | 0.288 |
| GOBP_RESPONSE_TO_ACID_CHEMICAL | 1.59 | 0.004 | 0.286 |
| GOBP_ARACHIDONIC_ACID_METABOLIC_PROCESS | 1.59 | 0.009 | 0.285 |
| GOBP_NEGATIVE_REGULATION_OF_NEURON_DEATH | 1.59 | 0.000 | 0.285 |
| GOBP_POSITIVE_REGULATION_OF_TYPE_I_INTERFERON_PRODUCTION | -1.59 | 0.006 | 1.000 |
| GOBP_RIBOSOMAL_SMALL_SUBUNIT_ASSEMBLY | -1.59 | 0.016 | 1.000 |
| GOCC_PROTEIN_COMPLEX_INVOLVED_IN_CELL_ADHESION | -1.59 | 0.015 | 1.000 |
| GOBP_MEMORY | 1.59 | 0.000 | 0.288 |
| GOCC_MITOCHONDRIAL_PROTEIN_CONTAINING_COMPLEX | -1.59 | 0.001 | 1.000 |
| GOCC_STEREOCILIUM_BUNDLE | 1.59 | 0.003 | 0.290 |
| GOBP_REGULATION_OF_DOUBLE_STRAND_BREAK_REPAIR_VIA_HOMOLOGOUS_RECOMBINATION | -1.59 | 0.006 | 1.000 |
| GOBP_NEGATIVE_REGULATION_OF_UBIQUITIN_PROTEIN_TRANSFERASE_ACTIVITY | -1.59 | 0.014 | 1.000 |
| GOBP_GLYCOPROTEIN_CATABOLIC_PROCESS | 1.58 | 0.026 | 0.289 |
| GOBP_RESPONSE_TO_FOLLICLE_STIMULATING_HORMONE | 1.58 | 0.049 | 0.288 |
| GOBP_POSITIVE_REGULATION_OF_VIRAL_GENOME_REPLICATION | -1.58 | 0.011 | 1.000 |
| GOBP_RESPONSE_TO_STARVATION | 1.58 | 0.000 | 0.287 |
| GOMF_CARBOHYDRATE_CATION_SYMPORTER_ACTIVITY | 1.58 | 0.041 | 0.288 |
| GOMF_BILE_ACID_TRANSMEMBRANE_TRANSPORTER_ACTIVITY | 1.58 | 0.050 | 0.287 |
| GOBP_VACUOLAR_TRANSPORT | 1.58 | 0.004 | 0.285 |
| GOMF_DNA_POLYMERASE_BINDING | -1.58 | 0.027 | 1.000 |
| GOBP_SPERM_FLAGELLUM_ASSEMBLY | 1.58 | 0.018 | 0.285 |
| GOBP_DNA_SYNTHESIS_INVOLVED_IN_DNA_REPAIR | -1.58 | 0.014 | 1.000 |
| GOBP_REGULATION_OF_LIPID_KINASE_ACTIVITY | -1.58 | 0.015 | 1.000 |
| GOBP_NEGATIVE_REGULATION_OF_GENE_EXPRESSION_EPIGENETIC | -1.58 | 0.006 | 1.000 |
| GOBP_NEGATIVE_REGULATION_OF_CYTOKINE_PRODUCTION_INVOLVED_IN_INFLAMMATORY_RESPONSE | 1.58 | 0.033 | 0.285 |
| GOBP_APPENDAGE_MORPHOGENESIS | -1.58 | 0.003 | 1.000 |
| GOBP_NEGATIVE_REGULATION_OF_AXONOGENESIS | 1.58 | 0.013 | 0.288 |
| GOMF_METHYL_CPG_BINDING | -1.58 | 0.016 | 1.000 |
| GOBP_REGULATION_OF_OLIGODENDROCYTE_DIFFERENTIATION | -1.57 | 0.010 | 1.000 |
| GOCC_INTRINSIC_COMPONENT_OF_NUCLEAR_INNER_MEMBRANE | -1.57 | 0.021 | 1.000 |
| GOBP_EMBRYONIC_HINDLIMB_MORPHOGENESIS | -1.57 | 0.030 | 1.000 |
| GOBP_MAINTENANCE_OF_CELL_NUMBER | -1.57 | 0.003 | 1.000 |
| GOBP_RESPONSE_TO_OXIDATIVE_STRESS | 1.57 | 0.000 | 0.295 |
| GOBP_CELLULAR_RESPONSE_TO_CADMIUM_ION | 1.57 | 0.017 | 0.293 |
| GOBP_REGULATION_OF_CENTRIOLE_REPLICATION | -1.57 | 0.023 | 1.000 |
| GOBP_HEAT_GENERATION | 1.57 | 0.045 | 0.297 |
| GOMF_POLY_PYRIMIDINE_TRACT_BINDING | -1.57 | 0.017 | 1.000 |
| GOBP_SUPEROXIDE_METABOLIC_PROCESS | 1.57 | 0.016 | 0.295 |
| GOBP_PROTEIN_LOCALIZATION_TO_NUCLEUS | -1.57 | 0.001 | 1.000 |
| GOBP_RESPONSE_TO_ARSENIC_CONTAINING_SUBSTANCE | 1.57 | 0.025 | 0.298 |
| GOBP_CELLULAR_RESPONSE_TO_MECHANICAL_STIMULUS | 1.57 | 0.012 | 0.298 |
| GOMF_HISTONE_METHYLTRANSFERASE_ACTIVITY | -1.57 | 0.020 | 1.000 |
| GOBP_BLASTOCYST_DEVELOPMENT | -1.57 | 0.004 | 1.000 |
| GOBP_RAB_PROTEIN_SIGNAL_TRANSDUCTION | 1.57 | 0.030 | 0.298 |
| GOBP_HISTONE_H3_K4_METHYLATION | -1.57 | 0.010 | 1.000 |
| GOBP_TORC1_SIGNALING | 1.56 | 0.018 | 0.297 |
| GOBP_POSITIVE_REGULATION_OF_TELOMERE_CAPPING | -1.56 | 0.045 | 1.000 |
| GOMF_RETINOIC_ACID_BINDING | -1.56 | 0.031 | 1.000 |
| GOCC_PROTEASOME_ACCESSORY_COMPLEX | -1.56 | 0.027 | 1.000 |
| GOMF_VITAMIN_TRANSMEMBRANE_TRANSPORTER_ACTIVITY | -1.56 | 0.020 | 1.000 |
| GOBP_ROOF_OF_MOUTH_DEVELOPMENT | -1.56 | 0.007 | 1.000 |
| GOBP_RESPONSE_TO_REACTIVE_OXYGEN_SPECIES | 1.56 | 0.000 | 0.297 |
| GOCC_ORGANELLE_ENVELOPE_LUMEN | -1.56 | 0.007 | 1.000 |
| GOMF_HYDROLASE_ACTIVITY_ACTING_ON_CARBON_NITROGEN_BUT_NOT_PEPTIDE_BONDS_IN_CYCLIC_AMIDES | -1.56 | 0.033 | 1.000 |
| GOBP_DEOXYRIBOSE_PHOSPHATE_METABOLIC_PROCESS | -1.56 | 0.022 | 1.000 |
| GOBP_INNER_MITOCHONDRIAL_MEMBRANE_ORGANIZATION | -1.56 | 0.010 | 1.000 |
| GOBP_NEGATIVE_REGULATION_OF_VIRAL_ENTRY_INTO_HOST_CELL | -1.56 | 0.020 | 1.000 |
| GOBP_HISTONE_H4_ACETYLATION | -1.56 | 0.003 | 1.000 |
| GOBP_POSITIVE_REGULATION_OF_LEUKOCYTE_CHEMOTAXIS | 1.56 | 0.004 | 0.303 |
| GOMF_INTRAMOLECULAR_TRANSFERASE_ACTIVITY | -1.56 | 0.028 | 1.000 |
| GOBP_AXON_EXTENSION | 1.56 | 0.003 | 0.304 |
| GOBP_REGULATION_OF_CENTROSOME_CYCLE | -1.56 | 0.018 | 1.000 |
| GOBP_CELLULAR_RESPONSE_TO_EXTRACELLULAR_STIMULUS | 1.56 | 0.000 | 0.304 |
| GOBP_NEGATIVE_REGULATION_OF_DNA_REPLICATION | -1.56 | 0.015 | 1.000 |
| GOBP_OLEFINIC_COMPOUND_BIOSYNTHETIC_PROCESS | 1.56 | 0.033 | 0.303 |
| GOMF_CALCIUM_ACTIVATED_CATION_CHANNEL_ACTIVITY | 1.56 | 0.033 | 0.303 |
| GOBP_PYRIMIDINE_NUCLEOSIDE_TRIPHOSPHATE_BIOSYNTHETIC_PROCESS | -1.56 | 0.021 | 1.000 |
| GOBP_POSITIVE_REGULATION_OF_EPITHELIAL_CELL_APOPTOTIC_PROCESS | 1.55 | 0.038 | 0.303 |
| GOCC_PROTEASOME_COMPLEX | -1.55 | 0.017 | 1.000 |
| GOBP_NEGATIVE_REGULATION_OF_ENDOPLASMIC_RETICULUM_STRESS_INDUCED_INTRINSIC_APOPTOTIC_SIGNALING_PATHWAY | -1.55 | 0.033 | 1.000 |
| GOMF_TRANSLATION_INITIATION_FACTOR_ACTIVITY | -1.55 | 0.016 | 1.000 |
| GOCC_DYNEIN_AXONEMAL_PARTICLE | -1.55 | 0.041 | 1.000 |
| GOMF_EXODEOXYRIBONUCLEASE_ACTIVITY | -1.55 | 0.026 | 1.000 |
| GOMF_ISOPRENOID_BINDING | -1.55 | 0.024 | 1.000 |
| GOMF_STEROL_TRANSPORTER_ACTIVITY | 1.55 | 0.027 | 0.315 |
| GOBP_CEREBELLAR_CORTEX_DEVELOPMENT | -1.55 | 0.017 | 1.000 |
| GOBP_NEGATIVE_REGULATION_OF_ORGANELLE_ORGANIZATION | -1.55 | 0.001 | 1.000 |
| GOBP_REGULATION_OF_DNA_DAMAGE_CHECKPOINT | -1.55 | 0.036 | 1.000 |
| GOBP_LOW_DENSITY_LIPOPROTEIN_PARTICLE_CLEARANCE | 1.55 | 0.037 | 0.315 |
| GOCC_PROTEASOME_REGULATORY_PARTICLE | -1.55 | 0.035 | 1.000 |
| GOBP_POSITIVE_REGULATION_OF_STEM_CELL_PROLIFERATION | -1.55 | 0.038 | 1.000 |
| GOBP_DNA_TEMPLATED_TRANSCRIPTION_ELONGATION | -1.55 | 0.006 | 1.000 |
| GOBP_MICROTUBULE_ORGANIZING_CENTER_LOCALIZATION | -1.55 | 0.024 | 1.000 |
| GOBP_NEURON_DEATH | 1.55 | 0.000 | 0.317 |
| GOBP_NEGATIVE_REGULATION_OF_MYOTUBE_DIFFERENTIATION | -1.55 | 0.033 | 1.000 |
| GOBP_MITOPHAGY | 1.54 | 0.038 | 0.317 |
| GOBP_PEPTIDYL_ARGININE_METHYLATION | -1.54 | 0.042 | 1.000 |
| GOMF_PROTEIN_METHYLTRANSFERASE_ACTIVITY | -1.54 | 0.006 | 1.000 |
| GOBP_REGULATION_OF_PROTEIN_LOCALIZATION_TO_NUCLEUS | -1.54 | 0.004 | 1.000 |
| GOBP_AXONEME_ASSEMBLY | 1.54 | 0.007 | 0.327 |
| GOBP_POSITIVE_REGULATION_OF_LEUKOCYTE_MIGRATION | 1.54 | 0.009 | 0.325 |
| GOMF_PHOSPHATIDIC_ACID_BINDING | -1.54 | 0.025 | 1.000 |
| GOBP_MICROTUBULE_DEPOLYMERIZATION | -1.54 | 0.028 | 1.000 |
| GOBP_POSITIVE_REGULATION_OF_MITOCHONDRIAL_TRANSLATION | -1.54 | 0.030 | 1.000 |
| GOBP_NEGATIVE_REGULATION_OF_MUSCLE_CELL_APOPTOTIC_PROCESS | 1.54 | 0.012 | 0.327 |
| GOBP_REGULATION_OF_DNA_STRAND_ELONGATION | -1.54 | 0.047 | 1.000 |
| GOBP_DOSAGE_COMPENSATION | -1.54 | 0.034 | 1.000 |
| GOMF_TRNA_SPECIFIC_RIBONUCLEASE_ACTIVITY | -1.54 | 0.038 | 1.000 |
| GOCC_TRANSFERASE_COMPLEX_TRANSFERRING_PHOSPHORUS_CONTAINING_GROUPS | -1.54 | 0.000 | 1.000 |
| GOBP_OOCYTE_MATURATION | -1.54 | 0.038 | 1.000 |
| GOCC_MICROTUBULE | -1.54 | 0.000 | 1.000 |
| GOMF_PROTEIN_CARRIER_CHAPERONE | -1.53 | 0.033 | 1.000 |
| GOBP_NEURON_CELLULAR_HOMEOSTASIS | 1.53 | 0.045 | 0.331 |
| GOMF_CADHERIN_BINDING | -1.53 | 0.000 | 1.000 |
| GOBP_NEGATIVE_REGULATION_OF_SYSTEMIC_ARTERIAL_BLOOD_PRESSURE | -1.53 | 0.039 | 1.000 |
| GOBP_REGULATION_OF_CELL_MIGRATION_INVOLVED_IN_SPROUTING_ANGIOGENESIS | 1.53 | 0.012 | 0.335 |
| GOBP_NUCLEOSIDE_TRIPHOSPHATE_BIOSYNTHETIC_PROCESS | -1.53 | 0.010 | 1.000 |
| GOBP_COGNITION | 1.53 | 0.000 | 0.334 |
| GOBP_BONE_MORPHOGENESIS | -1.53 | 0.011 | 1.000 |
| GOBP_HISTONE_H3_K9_METHYLATION | -1.53 | 0.026 | 1.000 |
| GOBP_HISTONE_DEACETYLATION | -1.53 | 0.006 | 1.000 |
| GOBP_CELLULAR_RESPONSE_TO_CORTICOSTEROID_STIMULUS | 1.53 | 0.023 | 0.335 |
| GOBP_NEGATIVE_REGULATION_OF_INTRINSIC_APOPTOTIC_SIGNALING_PATHWAY_BY_P53_CLASS_MEDIATOR | 1.53 | 0.048 | 0.334 |
| GOBP_PROTEIN_HETEROOLIGOMERIZATION | -1.53 | 0.026 | 1.000 |
| GOBP_REGULATION_OF_CELL_CYCLE_G1_S_PHASE_TRANSITION | -1.53 | 0.003 | 1.000 |
| GOBP_ORGANELLE_TRANSPORT_ALONG_MICROTUBULE | 1.53 | 0.013 | 0.334 |
| GOBP_EMBRYONIC_SKELETAL_JOINT_DEVELOPMENT | -1.53 | 0.037 | 1.000 |
| GOBP_MUSCLE_CELL_APOPTOTIC_PROCESS | 1.53 | 0.020 | 0.333 |
| GOBP_POSITIVE_REGULATION_OF_REACTIVE_OXYGEN_SPECIES_METABOLIC_PROCESS | 1.53 | 0.011 | 0.331 |
| GOCC_SMALL_RIBOSOMAL_SUBUNIT | -1.53 | 0.016 | 1.000 |
| GOBP_NEGATIVE_REGULATION_OF_EPITHELIAL_CELL_DIFFERENTIATION | -1.53 | 0.021 | 1.000 |
| GOBP_REGULATION_OF_MEIOTIC_NUCLEAR_DIVISION | -1.53 | 0.046 | 1.000 |
| GOMF_BETA_TUBULIN_BINDING | 1.53 | 0.030 | 0.331 |
| GOCC_PHOTORECEPTOR_CONNECTING_CILIUM | 1.53 | 0.024 | 0.331 |
| GOBP_ASTROCYTE_ACTIVATION | 1.53 | 0.049 | 0.329 |
| GOBP_GMP_METABOLIC_PROCESS | -1.53 | 0.023 | 1.000 |
| GOBP_REGULATION_OF_TRANSLATIONAL_INITIATION | -1.53 | 0.013 | 1.000 |
| GOCC_PROTEIN_ACETYLTRANSFERASE_COMPLEX | -1.53 | 0.011 | 1.000 |
| GOBP_CYTOCHROME_COMPLEX_ASSEMBLY | -1.52 | 0.033 | 1.000 |
| GOBP_HISTONE_H3_K4_TRIMETHYLATION | -1.52 | 0.043 | 1.000 |
| GOBP_REGULATION_OF_ACTIN_FILAMENT_BASED_MOVEMENT | -1.52 | 0.029 | 1.000 |
| GOCC_INTEGRIN_COMPLEX | -1.52 | 0.014 | 1.000 |
| GOBP_REGULATION_OF_ICOSANOID_SECRETION | 1.52 | 0.050 | 0.334 |
| GOBP_REGULATION_OF_ATP_DEPENDENT_ACTIVITY | -1.52 | 0.016 | 1.000 |
| GOBP_SEMAPHORIN_PLEXIN_SIGNALING_PATHWAY | 1.52 | 0.026 | 0.332 |
| GOBP_CELLULAR_RESPONSE_TO_ACID_CHEMICAL | 1.52 | 0.007 | 0.330 |
| GOBP_REGULATION_OF_MITOCHONDRIAL_GENE_EXPRESSION | -1.52 | 0.035 | 1.000 |
| GOBP_POSITIVE_REGULATION_OF_NEURON_DEATH | 1.52 | 0.003 | 0.329 |
| GOMF_CYTOSKELETAL_MOTOR_ACTIVITY | -1.52 | 0.007 | 1.000 |
| GOBP_NEGATIVE_REGULATION_OF_NEURON_PROJECTION_DEVELOPMENT | 1.52 | 0.004 | 0.330 |
| GOCC_MITOCHONDRIAL_SMALL_RIBOSOMAL_SUBUNIT | -1.52 | 0.032 | 1.000 |
| GOBP_DNA_DAMAGE_RESPONSE_SIGNAL_TRANSDUCTION_BY_P53_CLASS_MEDIATOR | 1.52 | 0.025 | 0.329 |
| GOBP_PURINE_NUCLEOSIDE_MONOPHOSPHATE_METABOLIC_PROCESS | -1.52 | 0.030 | 1.000 |
| GOMF_INWARD_RECTIFIER_POTASSIUM_CHANNEL_ACTIVITY | 1.52 | 0.043 | 0.327 |
| GOBP_REGULATION_OF_LEUKOCYTE_MIGRATION | 1.52 | 0.000 | 0.327 |
| GOBP_RESPONSE_TO_LEUKEMIA_INHIBITORY_FACTOR | -1.52 | 0.011 | 1.000 |
| GOBP_PROTEIN_IMPORT_INTO_MITOCHONDRIAL_MATRIX | -1.52 | 0.047 | 1.000 |
| GOBP_REGULATION_OF_URINE_VOLUME | -1.52 | 0.033 | 1.000 |
| GOBP_C21_STEROID_HORMONE_METABOLIC_PROCESS | 1.52 | 0.037 | 0.328 |
| GOBP_POSITIVE_REGULATION_OF_PROTEIN_LOCALIZATION_TO_NUCLEUS | -1.52 | 0.006 | 1.000 |
| GOBP_AXIS_ELONGATION | -1.52 | 0.031 | 1.000 |
| GOMF_TRANSLATION_REGULATOR_ACTIVITY_NUCLEIC_ACID_BINDING | -1.51 | 0.008 | 1.000 |
| GOBP_REGULATION_OF_PEPTIDYL_LYSINE_ACETYLATION | -1.51 | 0.008 | 1.000 |
| GOBP_REGULATION_OF_PROTEIN_ACETYLATION | -1.51 | 0.015 | 1.000 |
| GOBP_PROTEIN_LOCALIZATION_TO_VACUOLE | 1.51 | 0.029 | 0.333 |
| GOBP_NEGATIVE_REGULATION_OF_TELOMERE_MAINTENANCE_VIA_TELOMERE_LENGTHENING | -1.51 | 0.038 | 1.000 |
| GOBP_REGULATION_OF_FATTY_ACID_TRANSPORT | 1.51 | 0.046 | 0.331 |
| GOBP_PURINE_NUCLEOBASE_METABOLIC_PROCESS | -1.51 | 0.045 | 1.000 |
| GOBP_LUNG_MORPHOGENESIS | -1.51 | 0.016 | 1.000 |
| GOBP_MITOCHONDRIAL_DNA_REPLICATION | -1.51 | 0.049 | 1.000 |
| GOBP_POSITIVE_REGULATION_BY_HOST_OF_VIRAL_TRANSCRIPTION | -1.51 | 0.042 | 1.000 |
| GOBP_TELOMERE_CAPPING | -1.51 | 0.033 | 1.000 |
| GOBP_DNA_MODIFICATION | -1.51 | 0.011 | 1.000 |
| GOBP_POSITIVE_REGULATION_OF_CILIUM_ASSEMBLY | -1.51 | 0.029 | 1.000 |
| GOBP_RIBOSE_PHOSPHATE_BIOSYNTHETIC_PROCESS | -1.51 | 0.001 | 1.000 |
| GOBP_ESTABLISHMENT_OF_CELL_POLARITY | -1.51 | 0.007 | 1.000 |
| GOBP_REGULATION_OF_RECEPTOR_RECYCLING | 1.51 | 0.048 | 0.335 |
| GOMF_OXIDOREDUCTASE_ACTIVITY_ACTING_ON_NAD_P_H_QUINONE_OR_SIMILAR_COMPOUND_AS_ACCEPTOR | 1.51 | 0.016 | 0.334 |
| GOBP_POSITIVE_REGULATION_OF_HISTONE_H3_K4_METHYLATION | -1.51 | 0.037 | 1.000 |
| GOMF_MICROTUBULE_BINDING | -1.51 | 0.000 | 1.000 |
| GOMF_XENOBIOTIC_TRANSMEMBRANE_TRANSPORTER_ACTIVITY | 1.51 | 0.042 | 0.334 |
| GOBP_NEGATIVE_REGULATION_OF_BONE_REMODELING | 1.51 | 0.044 | 0.333 |
| GOBP_GLYCOSYL_COMPOUND_CATABOLIC_PROCESS | 1.51 | 0.030 | 0.331 |
| GOBP_POSITIVE_REGULATION_OF_TOR_SIGNALING | 1.51 | 0.026 | 0.330 |
| GOBP_GLYCEROL_ETHER_METABOLIC_PROCESS | -1.51 | 0.046 | 1.000 |
| GOBP_PHOTORECEPTOR_CELL_MAINTENANCE | 1.51 | 0.028 | 0.330 |
| GOMF_NUCLEAR_LOCALIZATION_SEQUENCE_BINDING | -1.51 | 0.045 | 1.000 |
| GOBP_NEGATIVE_REGULATION_OF_CELL_DIVISION | -1.51 | 0.042 | 1.000 |
| GOBP_CHRONIC_INFLAMMATORY_RESPONSE | -1.51 | 0.041 | 1.000 |
| GOCC_CHROMOCENTER | -1.51 | 0.038 | 1.000 |
| GOCC_STEREOCILIUM_TIP | 1.51 | 0.037 | 0.331 |
| GOBP_NEGATIVE_REGULATION_OF_I_KAPPAB_KINASE_NF_KAPPAB_SIGNALING | 1.51 | 0.036 | 0.330 |
| GOBP_MOTILE_CILIUM_ASSEMBLY | 1.51 | 0.012 | 0.329 |
| GOBP_NEGATIVE_REGULATION_OF_SMOOTH_MUSCLE_CELL_PROLIFERATION | 1.51 | 0.028 | 0.328 |
| GOMF_OXIDOREDUCTASE_ACTIVITY_ACTING_ON_A_SULFUR_GROUP_OF_DONORS | 1.50 | 0.006 | 0.327 |
| GOBP_ANTEROGRADE_AXONAL_TRANSPORT | 1.50 | 0.017 | 0.325 |
| GOBP_REGULATION_OF_TELOMERASE_ACTIVITY | -1.50 | 0.024 | 1.000 |
| GOBP_RESPONSE_TO_NERVE_GROWTH_FACTOR | -1.50 | 0.022 | 1.000 |
| GOBP_STEROL_METABOLIC_PROCESS | 1.50 | 0.004 | 0.325 |
| GOBP_NEGATIVE_REGULATION_OF_FATTY_ACID_BIOSYNTHETIC_PROCESS | 1.50 | 0.045 | 0.327 |
| GOMF_NUCLEOCYTOPLASMIC_CARRIER_ACTIVITY | -1.50 | 0.046 | 1.000 |
| GOBP_NEGATIVE_REGULATION_OF_TELOMERE_MAINTENANCE_VIA_TELOMERASE | -1.50 | 0.039 | 1.000 |
| GOBP_PROTEIN_LOCALIZATION_TO_LYSOSOME | 1.50 | 0.025 | 0.328 |
| GOBP_CELLULAR_PIGMENTATION | 1.50 | 0.033 | 0.327 |
| GOBP_MRNA_CATABOLIC_PROCESS | -1.50 | 0.001 | 1.000 |
| GOMF_OXIDOREDUCTASE_ACTIVITY_ACTING_ON_PAIRED_DONORS_WITH_INCORPORATION_OR_REDUCTION_OF_MOLECULAR_OXYGEN_REDUCED_FLAVIN_OR_FLAVOPROTEIN_AS_ONE_DONOR_AND_INCORPORATION_OF_ONE_ATOM_OF_OXYGEN | 1.50 | 0.023 | 0.326 |

**Supplementary Table 4.** Sequence of primers used for qPCR.

| **Gene** | **Forward primer (5'-3')** | **Reverse primer (5'-3')** |
| --- | --- | --- |
| HO-1 | ATTTCAGAAGGGCCAGGTGA | GGAAGTAGACAGGGGCGAAGA |
| NOQ1 | CCCCGGACTGCACCAGAGC | CTGCAGCAGCCTCCTTCATGGC |
| OSGIN1 | GCAGCAGATGATGCGTGAC | GGAGCCGATGAGGACGAG |
| 18s | CAGAAGGATGTAAAGGATGG | TATTTCTTCTTGGACACACC |
